# Supplementary material for: Early contact between late farming and pastoralist societies in southeastern Europe
Source: Nature. 2023 Jul 19;620(7973):358–65. doi: 10.1038/s41586-023-06334-8 (PMC10412445; doi:10.1038/s41586-023-06334-8)
Supplement: Supplementary file 1 — This file contains Supplementary Text 1–7, Fig. 1 and additional references. [file 41586_2023_6334_MOESM1_ESM.pdf]

nature portfolio

https://doi.org/10.1038/s41586-023-06334-8

Supplementary information

Early contact between late farming and pastoralist societies in southeastern Europe

In the format provided by the authors and unedited

SUPPLEMENTARY INFORMATION

Early contact between late farming and pastoralist societies in southeastern Europe

Sandra Penske[1,*], Adam B. Rohrlach[1,2], Ainash Childebayeva[1], Guido Gnecchi-Ruscone[1], Clemens Schmid[1], Maria A. Spyrou[1,3], Gunnar U. Neumann[1], Nadezhda Atanassova[4], Katrin Beutler[5], Kamen Boyadzhiev[6], Yavor Boyadzhiev[6], Igor Bruyako[7], Alexander Chohadzhiev[8], Blagoje Govedarica[8], Mehmet Karaucak[5], Raiko Krauss[9], Maleen Leppek[10], Igor Manzura[11], Karen Privat[12], Shawn Ross[13], Vladimir Slavchev[9], Adéla Sobotkova[14], Meda Toderaş[15], Todor Valchev[16], Harald Ringbauer[1], Philipp W. Stockhammer[1,10], Svend Hansen[5], Johannes Krause[1], Wolfgang Haak[1,*]

This PDF files includes

Supplementary Text

1. **Southeastern Europe**
    - 1.1 Ecological background
    - 1.2 Archaeological background
2. **Northwestern Black Sea Region**
    - 2.1 Ecological background
    - 2.2 Archaeological background
3. **Archaeological context of sites and newly reported individuals**
    - 3.1 **Southeastern Europe**
        - 3.1.1 Petko Karavelovo
        - 3.1.2 Pietrele
        - 3.1.3 Varna
        - 3.1.4 Yunatsite
    - 3.2 **Northwestern Black Sea Region**
        - 3.2.1 Kartal
        - 3.2.2 Majaky
        - 3.2.3 Usatove
4. **Testing for steppe-related ancestry in published CTC-associated individuals**
5. **Rationale for selecting source populations**
6. **Rationale for grouping of proximal source population in qpAdm**

7. **Intra- and inter-site relatedness using READ**

References

1. Southeastern Europe

1.1 Ecological background

During the Late Neolithic and the Copper Age, climatically optimal conditions prevailed. A continental climate with cold dry winters and moist early summers must have been the norm, which is reflected in the adaptation and choice of cultivated and exploited plants. The main attested species in the macro-botanical assemblage are einkorn and emmer wheat, barley, lentils, peas and bitter vetch. Collected fruits included raspberry, cornelian cherry, cherry plum, grapes and blackthorn.

The paleoenvironmental studies at tell Pietrele have provided evidence that the Danube did not exist in the form of a streamlined river as we know it today[13]. In addition to many side-arms, the Danube to the east of the city of Ruse was rather a lake that at times probably reached as far as the Black Sea.

This meant an almost inexhaustible supply of fish and mollusks for the inhabitants at this lake. In Pietrele for example, mainly carp and pike were fished, and to a lesser extent also catfish. While the older layers of this tell are mainly characterized by domesticated animals, especially pigs, but also cattle and sheep, in the younger layers half of the animals are wild game, indicating that the hunting of wild boar, red deer, wild horse and aurochs played a substantial role[95]. Comparable results of the palaeobotanical and palaeozoological investigations are also available from other tell settlements.

The end of the tell settlements has been linked, above all by Henrieta Todorova, to a rise in annual mean temperature that led to long-lasting droughts and forest fires and finally to a prolonged depopulation over several centuries[16].

1.2 Archaeological background

During the 5th and 4th millennia BCE, processes of far reaching technical and social change took place between the Balkans and the Caucasus, resulting in a complete transformation of Neolithic societies. Metal production was the most important innovation of the 5th millennium BCE. Copper was mined and smelted. The metal was used to make heavy axes, jewelry pins and small tools. In the cemetery of Varna on the Bulgarian Black Sea coast the oldest gold artifacts in Europe were found. Together with golden scepters and diadems, symbols of power to this day, which were also found in the cemetery, these finds mark the beginning of strong social inequalities and steep hierarchies[9,12]. The ^{14}C dates of the necropolis cover a 260-year period, beginning before ~4600 BCE and ending shortly after ~4400 BCE[58,96].

The cemetery belongs to one of settlements, that were built on the shore of Lake Varna, and which are submerged today. The site is eponymous for the so-called Varna culture.

Long-term excavations of the settlement mound Măgura Gorgana near Pietrele on the Lower Danube in southern Romania, 170 km inland from Varna, provided evidence for continuous occupation between ~4600 and 4250 BCE[96]. The settlement was a new foundation, probably part of an organised colonisation of the region. There is clear evidence for craft specialisation, division of labour, and a system of redistribution organised by a chief[97]. The duration of the Pietrele settlement, like the entire settlement system on the Lower Danube, lasted ~350 years until it collapsed. Pietrele is part of the so-called Gumelnița culture, a regional group of the Copper Age on the Eastern Balkan peninsula and common in southern Romania. Various regional archaeological cultural groups can be distinguished here between the Danube, the Aegean and the Black Sea.

Yunatsite is located near Pazardzik in western Bulgaria and is attributed to the Karanovo culture. This settlement mound ended as early as ~4400 BCE due to a violent attack and was not settled again until the Early Bronze Age after more than a thousand years[14].

After the collapse and abandonment of most Copper Age sites around 4250 BCE, evidence of settlement is largely lacking in the entire western Black Sea region for the following centuries. A sharp population decline can be postulated. Nevertheless, the Neolithic economy continued in the area of the Trypillia culture further east in the forest steppe, which was already organised in very large settlements with thousands of houses at the end of the 5th and the beginning of the 4th millennium BCE[18,46].

The 4th millennium BCE was marked by a remarkable accumulation of key innovations that were crucial for further development. In the field of metallurgy, the alloying of copper was significant, because with the addition of arsenic, the soft copper became hard bronze[99]. The alloy facilitated the development of new weapons such as daggers and swords. The introduction of the wheel and wagon was another key innovation. No less important were the beginnings of horse domestication and the breeding of the woolly sheep. All these innovations had far-reaching effects on the economy, armed conflicts and social organisation and thus spread rapidly across western Eurasia – often independently and not as a uniform package. Innovations were integrated into regional systems in different ways with different consequences. While cities and early states developed in Mesopotamia, villages and chiefdoms remained the predominant form of community organisation in Europe. Taken together, the 4th millennium BCE can be regarded as a time period during which the foundations of the modern technosphere were laid and new and pertaining social relations were installed[98].

The spread of variants of the Gumelniţa culture north of the Danube, and thus the spread of agriculture into the steppe zone during the middle of the 5th millennium BCE marks the beginning of the Eneolithic in the North-West Black Sea[99]. In lieu of these developments the North Pontic region and the Caucasus probably played a role at various levels. For example, the development of the oldest wheeled vehicles was postulated in the area of the Trypillia culture[22]. In the North Caucasus, the Maykop culture may have played an important role in the development of metal alloys. The famous Oshad Maykop burial mound harbored a set of arsenic bronze implements, of which a dagger is astonishingly 34.7 cm long[23]. This Maykop kurgan was one of the earliest tombs for one individual with a 100m diameter and 10m height that underpins the social upheavals at that time. The beginnings of the domestication of horses can also be linked with the (Steppe) Maykop culture[7]. Likewise, the development of the woolly sheep economy combined with extensive dairy production may have taken place here[8]. The east-west exchange of not only innovative products but also of technical know-how took place during the 4th millennium BCE via the northern Pontic steppe region. In the Dnepr-Azov region several cultural groups (Lower Mikhaylovka and others) with different traditions, burial customs and economic activities were involved[42].

At the end of the 4th millennium BCE, burial mounds with metal grave goods representing a social elite can also be found further west in southern Romania and in Bulgaria[24].

Archaeological evidence suggests that settlements during the subsequent Early Bronze Age (EBA), dated in the Eastern Balkans between 3200 and 2500 BCE became numerous again for the first time after the end of the tell settlements in the eastern Balkan region.

2. Northwestern Black Sea Region

2.1. Ecological background[100,101]

The northwestern Black Sea region is bordered on the west by the Danube and Prut River and on the east by the Tiligul River. The northern border runs along a line separating the steppe environment from the forest-steppe zone. From the south and southeast the region is bounded by the Black Sea. The area can be considered a separate territory with a specific combination of cultural traditions and independent trajectories of culture-historical developments. The southwestern part of the region is characterized by several Danubian freshwater lakes such as Cahul, Yalpug, Kotlabuch and Kitay, with a well-developed hydrographic network which is favorable for primitive/early agriculture. The northwest is distinguished by a hilly relief with extensive patches of broadleaf forest called *Codrii Tigheci*.

Along the Black Sea shore, flat lowlands with saltwater lakes and estuaries which stretch to the Azov Sea are predominant. The area north of the lowlands is a billowy plain with broad river valleys and vast steppe plateaus. The region between the Danube/Prut and the Dniester River is predominantly covered by the Budzhak herb steppe with a typical xerophytic and mesophytic vegetation. The steppe environment is extended further east of the Dniester and forests from the north spreading along the river valleys create an additional variety of existing ecotones. This junction of different ecological environments generates favorable conditions for diversified and coexisting economies during the Eneolithic and EBA including agriculture in river valleys, semi-mobile stockbreeding on steppe plateaus, and hunting and fishing. These exact conditions provide the background for the emergence of complex cultural entities such as the Cernavodă I and Usatove cultures at the end of the Eneolithic and in the beginning of the Bronze Age which were able to combine developed agriculture with distant stockbreeding.

2.2 Archaeological background

During the second half of the 7th millennium BCE, the Balkan-Carpathian region was settled by early farming communities which, in the 6th millennium BCE, moved further into the forest-steppe zone between the Prut and the Dniester, bringing new economic innovations into this region[102]. In the second half of the 6th millennium BCE early farmers associated with the Linearbandkeramik culture (LBK) settled in the forest zone of Central Europe, reaching the Paris Basin in the west, and occupying forest and forest-steppe territories east of the Carpathians up to the Southern Bug River and the Dnieper[103]. Almost simultaneously, a peculiar type of Neolithic culture, probably still based mainly on a hunting-gathering economy, co-existed in the eastern European steppes. However, hardly anything is known about the northwestern Black Sea region at this time. In fact, almost no traces of any Neolithic culture can be found in this area in the 7th and 6th millennia BCE, except for sparse remains of rare short-term camps of the Bug-Dniester culture east of the Dniester[104], and isolated finds of the Boian and LBK cultures in the area of Danubian lakes. It is possible that natural resources in the region were so abundant and variable that there was no necessity for local hunters and fishers to accept and transition into a new mode of life. This situation likely continued until the middle of the 5th millennium BCE.

The beginning of the Eneolithic in the North-West Black Sea region then coincided with the emergence of the Bolgrad-Aldeni variant of the Gumelniţa culture north of the Danube in the middle of the 5th millennium BCE, according to radiocarbon dates[105]. This event can be considered as an attempt of an agricultural spread into the steppe zone. The Gumelniţa culture

is characterized by their long-term substantial settlements with surface houses and fortifications along rivers permanently extending their cultural area further to the north. Most of the Gumelniţa culture settlements can be found around the Danubian lakes. However, after 150-200 years, farming settlements of the Gumelniţa culture in the steppe zone were suddenly abandoned in order to give way to new cultural phenomena in the second half of the 5th millennium BCE.

The Suvorovo group appeared in the North-West Black Sea region[106] and is usually considered the westernmost variety of the extensive Khvalynsk-Sredny Stog cultural entity. Cemeteries and isolated graves of the Suvorovo type are found all over the region from the Tiligul to the Danube. These groups' burial rites are characterized by a crouched position of the individual on their back, and an abundant use of ochre. Some cemeteries stand out by complex burial structures and lavish grave offerings (*e.g.,* Giurgiuleşti, Căinari). At the same time, there are cemeteries or isolated graves with simple burial pits and rather sparse grave goods (*e.g.*, Novoselskoe, Koshary, Novaya Dolina).

The development of the Suvorovo group was under strong influence of the Gumelniţa A2 and Cucuteni A-Trypillia B1 cultures which is documented by imported farming pottery, metal objects and some details of mortuary rituals. It is worth noting that despite the influence of farming cultures most of the steppe communities probably maintained the previous lifestyle of hunting and fishing although use of domestic animals in groups close to agricultural areas could also have been practiced.

Around 4200 BCE, the flourishing, largely agrarian Eneolithic cultures in the eastern Balkans suddenly collapsed, and the cultural development in this area entered into a latent phase, the so-called Balkan Dark Ages, which lasted for about 400-500 years. A similar situation, a so-called "steppe hiatus", can be found to some extent in the Black Sea steppe zone east of the Tiligul River[40]. The length of this hiatus is similar to the Balkan Dark Ages. Archaeological records in the North-West Black Sea region at the end of the 5th and beginning of the 4th millennia BCE also demonstrate apparent gaps in cultural development.

This "invisibility" of cultural development in steppe territories can be partly explained by the disappearance of agricultural ceramic imports during the time of the Cucuteni A-B-Trypillia B1/B2 culture. It can be theorized that communities of the Cucuteni-Trypillia culture initially avoided close contact with inhabitants of the steppe area during this period. However, further into the Eneolithic the Cucuteni-Trypillia culture potentially played an important role in the cultural transformation of the steppe zone.

Around 3800 BCE a series of new cultural phenomena arose in the eastern European steppes with the Lower Mikhailovka, Kvitynskaya, Dereivka and Stogovskaya cultures in the Lower Dnieper-Northern Azov region, the Konstantinovskaya culture in the Lower Don, and the Northern Donets Basin and burial sites of the Berezhnovka-Arkhara type in the Lower Volga area[41,107]. The new cultural entities demonstrate diversity not only in observed in the material culture but also in mortuary traditions unlike in the early Eneolithic period for which rather uniform burial customs are documented. Simultaneously, the Maykop cultural phenomenon appeared north of the Greater Caucasus Mountain range and influenced the adjacent and more distant steppe groups.

The eastern Balkans underwent similar cultural changes. South of the Lower Danube and in the eastern Thrace the Pevets[108,109] (Khontitsa) culture with its long-term settlements emerged, marking an agricultural resettlement of this area. Similarly, numerous habitation sites of the Cernavodă I culture appeared in Muntenia and Dobrudzha and other places in the North-West Black Sea region, which inherited to a large degree the material culture of the preceding Gumelniţa culture[25,109,110] (Extended Data Fig. S1e, f). Outside of the core area of the Cernavodă I culture, burial sites with pottery are the predominant remains in this area and evidence for a settlement is only found north of the Danube at Orlivka (Reni district of the Odesa County). The material record documents the development of an effective economic system combining agriculture and semi-sedentary pastoralism with a focus on small livestock. Such preference and adjustments in stockbreeding practices facilitated the adaptation to surrounding steppe environments on elevated plateaus and gained more significance for further economic and social developments in the northwestern Black Sea region.

Another source of a strong cultural influence was located in the area of the Cucuteni B-Trypillia C1 culture with its mega-sites and technological achievements. Many steppe burials of this period contain painted vessels of this farming culture, and settlements with a hybrid material culture appeared on the northern periphery of the Cernavodă I culture. It is also suggested that the Cucuteni-Trypillia culture started to venture further into steppe areas. The archaeological evidence suggests that steppe territories were simultaneously controlled by newcomers from farming cultural background and local steppe inhabitants.

The cultural development in the eastern part of the northwestern Black Sea region advanced further and culminated with the emergence of the Usatove culture in the middle of the 4th millennium BCE[111]. Here, a complex organized society with hierarchical structures, rigid social gradations, extensive external relations and an elite culture is represented. Leaders of this society effectively utilized the technological innovations and social experiences of earlier

generations in order to create a well-balanced economic system and resistant social structures well accommodated to the local steppe environment. Traditions of the later Cernavodă I culture were maintained in the area of the Danubian lakes and in the littoral zone of the Dniester-Danube interfluve. They, in many relations, correspond to basic attributes of the Usatove culture but do not demonstrate the same level of complexity expressed in burial customs and material traits.

In the last quarter of the 4th millennium BCE the northwestern Black Sea region experienced a new cultural transformation. The Usatove culture disintegrated in the eastern part of the region whereas the last manifestations of the Cernavodă I culture faded finally in its western part. A new cultural phenomenon designated as Zhivotilovka-Volchansk type with apparent interregional character emerged in the eastern European steppes[112]. Burial sites of this type appeared not only in the former areas of the Usatove and Cernavodă I culture but also spread across vast steppe territories from the Northern Caucasus to the Carpathians and absorbed different cultural traditions of the Usatove culture, late Trypillia communities, the Maykop-Novosvobodnaya entity and North Pontic steppe groups. Disintegration of earlier cultural borders probably created favorable conditions for a fast expansion of the Yamnaya culture at the very end of the 4th millennium BCE which then formed a specific cultural variant in the northwestern Black Sea region designated as the Budzhak culture.

3. Archaeological context of sites and newly reported individuals

(In alphabetical order)

3.1 **Southeastern Europe**

3.1.1 Petko Karavelovo, Bulgaria

N 43.30°; E 25.64nn

Excavations by the Regional Museum of History – Veliko Tarnovo.

The village of Petko Karavelovo (known before as Sashevo until 1993, and Odaite until 1950) is in the central part of north Bulgaria, and is situated 27 km northwards from the town of Veliko Tarnovo.

The archaeological tell site stands 1.5 km south from the village and 1.5 km westwards from the modern bed of the Yantra River, in visual proximity to the international E 85 road. It is relatively small – rising just 3 meters from the terrain, but its cultural layer is 4.10 m thick. The tell is situated in the middle of the vast valley of Yantra River, but within an older bed, rather than on a terrace. The river now flows at the eastern end of the valley but only after the construction of corrections and a system of dykes in the second half of the last century. Only after the corrections, the Yantra River had been periodically changing its course, and the land between the river and the mound was gained for agriculture. The altitude at the highest point of the mound is 48.6 m. The altitude of the river bed today is almost the same as that of the early layers. Petko Karavelovo, although geographically belonging to the Yantra's downstream, is almost in the middle of the Danube plain at about 40 km away, both from the Danube and the Balkan Mountains. The site is the only known Chalcolithic tell so close to the Yantra river itself, whereas all others in the region are situated on its tributaries. The settlement itself was frequently flooded and later, the cultural layer has been repeatedly exposed to water due to the proximity of the river[113].

The regular excavations started in 2009. Up until now, seventeen occupational levels are documented. Based on ceramic and architectural dynamics, as well as stratigraphic sequences, they are separated in three periods: Petko Karavelovo A (A1-A4) – Early Chalcolithic 5000-4800 BCE; Petko Karavelovo B (B1; B1-2; B2) – Middle Chalcolithic 4800-4650 BCE; Petko Karavelovo (C1-C10) – Late Chalcolithic 4650-4350 BCE. Architectural structures from the last two phases, PK-C9 and PK-C10, were not recorded on the site, due to erosion, agriculture, and flooding. They are established only by materials and in negative features. The 2018 excavation campaign offered a multiple burial of five individuals in extremely unusual and uncharacteristic positions[114].

The burial pit (Feature E 11) is slightly oval, with the long axis in an east-to-west direction. The length is 2.03 m, and the width (north-south) is 1.36 m. The order of the skeletons is as follows: individual C first fell into the pit, followed by D, and then E, covered by B and the last individual was A. The pose of all five individuals is atypical for any burial ritual known during this time period. They are all piled on top of each other. The pit was excavated in the burnt ruins of a house (feature E5) very soon after its destruction. This house belongs to the PK-C3 phase. The destruction of the burnt house and the pit were sealed by the massive clay floor of the following house (feature E3), belonging to the next PK-C4 phase of the site.

The anthropological analysis shows that all five individuals were male, aged from 17-20 years (Juvenis (individual B)) up to 35-40 years (Adultus (individual C)). The average value

for stature ranges from 157.81 cm (individual A) to 167.53 cm (individual E). All of the individuals, except C, lay with the anterior surface of the bones to the soil, i.e., had been laid facing the soil. Only individual C (the lowest one) laid with his back towards the bottom of the pit. Three of the individuals have their heads to the east (individuals C, D and A – the first two, and the uppermost one). The other two, E and B have their heads turned westwards. Three of the individuals (C, D and A) showed signs of caries and in three cases (C, B and A) dental calculus was observed. Individual E has a biradicular lower left mandibular canine, which is a rare anatomical variation. All individuals, except individual B, show a lateral epicondylitis of the two shoulder bones which results from a chronic loading of the two upper limbs. On individual C *spina bifida oculta* is established in the area of IV and V sacral vertebrae. All five individuals have traces of multiple and heavy violent impact on the skull. The blows occured in the presence of soft tissues and can be considered the cause of death. The strikes can be divided into two groups - oval and irregular. There are some of the peculiarities in the upper limbs' positions, especially in individuals C and D, suggesting that they may not have been fully dead at the moment they had fallen into the pit (the so-called "*Cadaveric spasm*"). The nature of the injuries, trauma most often to the head, with multiple injuries, indicates targeted violence against the five individuals, exercised with particular cruelty since the frontal traumas indicate violence against individuals in a helpless condition. The treatment of the bodies, rather the lack of any burial rites and the complete absence of inventory, speak of a hostile attitude towards these individuals. In some societies there is a special attitude towards "their" dead. However, in this specific situation, such an attitude is lacking. In contrast, the individuals bear marks of manifestations of particular cruelty and the "burial" itself seems to demonstrate the superiority of the "buriers" over the buried. The place of the "burial" not just within the settlement, but specifically within the destroyed, burnt house also seems not accidental. Apparently the "buriers" did not accept the "buried" as their own, whose place, according to usual practice, is in the cemetery. However, there is a clear desire to keep the bodies within the settlement, rather than simply getting rid of the corpses. There is evidence that lumps from the burnt house were used to strike the heads and bodies of the buried and therefore somehow connect them to the events that led to the house's destruction and burning. That is why a hypothesis about the execution of captured outsiders and their burial within the settlement, as a "trophy" in the common memory of the tell dwellers, cannot be rejected. The positions of the skeletons suggest that the execution took place in the immediate vicinity of the pit, the bodies being pushed into it before the final onset of death as in the case of individual C and D. Soon after the pit was filled in, the floor of the next house was built on top. This resulted in relatively

hollow skulls of all individuals. The massive clay floor sealed the pit from external influences, and the burnt clay from the dwelling's destruction helped to avoid the processes that lead to the filling of the skulls with earth. To what extent the five buried individuals were "foreign" can only be said after more research of the necropolis of the tell site.

Human skeletal remains from Petko Karavelovo included in this study:

PTK003. Individual C. Belongs to the genetic PTK_CA cluster.
Area E, sq. 77, feature E11. Dating of the skeleton: 4674-4461 calBCE (5721±24 BP, MAMS-51580)

PTK004. Individual D. Belongs to the genetic PTK_CA cluster.
Area E, sq. 77, feature E11. Dating of the skeleton: 4656-4455 calBCE (5708±28 BP, MAMS-38611)

PTK005. Individual E. Belongs to the genetic PTK_CA cluster.
Area E, sq. 77, feature E11.

3.1.2 Pietrele, Romania

N 44.06°, E 26.16°

Excavated by the German Archaeological Institute, Berlin, the Institute for Archaeology "Vasile Pârvan" of the Romanian Academy of Sciences, Bucharest and the Institute for Geography of the Goethe University, Frankfurt/Main

The settlement mound "Măgura Gorgana" is situated approximately 7 km north of the Danube River, near the village of Pietrele, jud. Giurgiu, Romania. The archaeological research at the site was initiated during the mid-1940s, when Dumitru Berciu excavated soundings at the eminence of the mound and distinguished multiple settlement layers associated with the Gumelniţa Culture. Since 2002, the fieldwork at the site has been continued by an international team of researchers as part of a collaboration between the Eurasia Department of the German Archaeological Institute in Berlin, the Institute for Archaeology "Vasile Parvan" of the Romanian Academy of Sciences in Bucharest and the Institute for Geography of the Goethe University in Frankfurt/Main.

The landscape surrounding the settlement preserved its wetland traits until the 1960s, when the entire floodplain was drained by the Romanian government in order to boost

agricultural production in the region. This wetland habitat is the relic of a large paleo-lake, which extended from the city of Giurgiu and stretched towards the west, through which the Danube River flowed eastwards the Black Sea. The lake must have been an almost inexhaustible food resource for the settlers of the region during the late 6th and 5th millennia BCE, as indicated by large quantities of fish bones and shells from the settlement layers at Pietrele. Furthermore, the lake connected the site with many other settlements across its shores, allowing access between sites via boat.

The entire duration of the Copper Age settlement on the mound at Pietrele (excluding the earlier Late Neolithic features around the settlement mound) can be tentatively dated between 4673-4457 calBCE (MAMS-19665, 95.4% probability) or 4672-4456 calBCE (MAMS-27452, 95.4% probability) and 4359-4241 calBCE (Bln-5721, 95.4% probability), or most likely between the 47th to 43rd centuries BC. Although the results of a small number of conventional charcoal samples do extend towards the 42nd/41st centuries, we believe that this discrepancy is due to the relatively flat plateau on the ^{14}C calibration curve at the end of the 5th millennium BCE.

A village several hundred years older at the same location was probably only recognizable as deserted ruins by the time the first generation of Copper Age settlers arrived at the lakeside. Although there are no formal Late Neolithic burials at the site, isolated fragments of bone belonging to a female individual from this period were recovered within a pit (PIE039). The Late Neolithic remains underneath the Copper Age strata at the settlement mound, as well as those in the surrounding area, can be dated within the 53rd to 51st centuries BCE and, accordingly, they are approximately 500 years older than the earliest Copper Age features at Pietrele.

The settlement mound was formed by repeated construction of new structures over the remains of older ones that had been abandoned, destroyed, or burnt down. The remains of a two-story structure are an exceptional find in Pietrele. Besides an almost complete inventory of household items preserved within, the structure also contained the charred bones of its inhabitants; nine individuals of various ages who met their demise by a sudden and deadly fire. The remains of the structure, together with the bodies of its inhabitants, were buried under a thick layer of sand and clay before the beginning of the construction of a new building over the same plot. This particular practice of filling and burying the abandoned and destroyed buildings can be observed in almost all other structures in Pietrele as well. Eventually, in a relatively short time span of 300 to 400 years, the settlement mound rose up to 11 meters above its surroundings. Such an imposing stratigraphy is seemingly unique in southeastern Europe and,

accordingly, Măgura Gorgana will be an important reference point for chronology as well as for future research into the development of economic strategies during the 5th millennium BCE.

From around 4450 BCE, the settlement grew in size and an extensive flat settlement with numerous additional houses emerged in the immediate vicinity of the settlement mound. Concurrently, we observed an expansion of economic activities at the site, as well as a notable increase in the volume of finds and accumulated material culture.

Division of labor among the inhabitants and specialization of households in certain crafts and economic activities is clearly recognizable in Copper Age Pietrele. For instance, the households excavated in the area of Trench B yielded a large number of tools for textile production: two of the oldest looms were found in this area *in situ*. The uninterrupted sequence of eight houses in Trench F, on the other hand, indicates a specialization in hunting and fishing, which was passed on over several generations. Here, for the first time, a family tradition developed whereby the children learned what their parents practiced as a profession. Ceramic vessels used by the inhabitants of Pietrele were likewise produced on a large scale by specialized potters. Further evidence of specialist-produced pottery can be seen in ceramics with intricate graphite decorations that require years of mastery over this technique, as well as a high degree of skill and hand-eye coordination. Large storage vessels, such as pithoi, must have also been made by specialized craftspeople. The largest such pithoi vessel from Pietrele has a volume of around 400 liters. This particular model of specialized household production based on a division of labor may be explained by a "redistributive system" in which an authority, such as a chief, would oversee and organize production, collect the resulting surplus, redistribute it among the households, and claim fees for doing so. The surplus was organized by order and certainly to no small extent under coercion.

The inhabitants of Pietrele certainly took part in long-distance trade and exchange. Such products include numerous flint blades. From about 4450 BCE onwards, the use of metal tools became much more commonplace at the site. Simple metal awls inserted into handles made of bone served as one of the first universal tools. A naturally occurring lead ore, galena, was smelted in small biconical vessels – the oldest evidence of this practice in southeastern Europe. Spondylus shells, fashioned into arm rings and beads, originate from the Aegean Sea. Numerous smaller and larger anthropomorphic statuettes and rich assemblages of decorated food-serving vessels suggest that Pietrele was also a center for feasts, festivals and commensal gatherings.

The entire settlement was burnt down around 4250 BCE and was never again rebuilt. The other settlements along the Lower Danube were also abandoned around roughly the same time. Many reasons are conceivable for the desolation of the region: Perhaps the natural

resources at the lake were exhausted or violent conflicts engulfed the area. In any case, there are very few archaeological traces of subsequent settlement in the region for several hundred years.

Archaeological human remains in Pietrele

The following catalog comprises the sample of human skeletal remains tested for ancient DNA preservation and the contexts in which they were found. Most of the sampled material derives from the graves situated between the flat settlement and as single finds or depositions from both the settlement mound and the flat settlement. Formal burials have not been observed in the settlement mound so far. Part of this study were the single bones and depositions, mostly found in the areas between the houses, middens, and fill layers between the structures. Such fills were constructed in order to bury the remains of abandoned structures and simultaneously serve as foundations for new constructions to be built on the same plots. Large volumes of soil, sand, and clay were brought from the vicinity of the settlement mound, including the area of the flat settlement. For this reason, most of the human bones found in such fill layers produced older ^{14}C dates than the samples taken from the houses on the same level or below them. Such samples include: P05B123 (PIE026); P08B108 (PIE034); P08B114 (PIE033); P11F881 (PIE029); P12B208 (PIE017); P15B730.

There is also a case of a mass burial in Pietrele. The bodies of five individuals, as well as single bones from other individuals, were deposited inside a narrow pit without any care given to position and orientation. Among them, the bodies of two male individuals (P11L273 (PIE004 and PIE015)) and three female individuals (P11L273 (PIE012, PIE035), P11L293-D (PIE048)) were analyzed. At the moment of their death, these individuals must have been in miserable condition, most probably due to hard physical work. Here, we get a rare glimpse into the lower end of society, perhaps slaves, from an early phase of the Copper Age settlement.

A huge shell midden was excavated during the course of two campaigns in the area of Trench L to the north of the settlement mound[115]. This distinctive midden contained roughly 26 kg of shells, as well as disarticulated bones and single skulls of at least three individuals (P10L234 (PIE041); P12L006 (PIE050); P18 F708 (PIE060)).

Between 2017 and 2019, the youngest domestic structure of the outer settlement so far was excavated at the foot of the western slope of the mound. A major surprise was the presence of contracted graves for three male individuals, including one child (P18 W629 (PIE057)) and two adults (P18 W650 (PIE054) and P18 W642 (PIE058)). These graves were "built" into the

foundation of the west wall of the structure. Genetic studies revealed that the adults within the graves had a second-degree relationship to each other.

The grave of a 35 to 45-year-old man (P11N705 (PIE007)) associated with the Late Copper Age Cernavodă Culture is situated to the west of the settlement mound and presents the earliest archaeological evidence of further activities at the site after the abandonment of the Copper Age settlement. He was accompanied by his dog and dagger, which were buried together with him[116]. Another grave dating to the Early Bronze Age comes from the same area (P16N513 (PIE078)).

Human skeletal remains from Pietrele included in this study:

PIE003. P11P104. Chalcolithic. Belongs to the genetic PIE_CA cluster.
Female individual, 25-35 years old. Grave found in the flat settlement, northeast of the tell. Bones are preserved in good condition. No pit visible. Orientated west-east, contracted on its right side, facing south. No grave goods, a grinding stone lies at her feet. Dating of the skeleton: 4486-4353 calBCE (5586±25 BP, MAMS-47794)[117].

PIE004. P11L273. Chalcolithic. Belongs to the genetic PIE_CA cluster.
Male individual. Mass grave found in the flat settlement, north of the tell. Bones were preserved in good condition. Dating of the skeleton: 4701-4544 calBCE (5770±26 BP, MAMS-47795)[117].

PIE005. P13T210. Chalcolithic. Belongs to the genetic PIE_CA cluster.
Male individual, 4-5 years old. Area with human remains found in the flat settlement north of the tell. Two children. The almost complete skeleton was found partially in anatomical position in contracted position on the right side, oriented southwest-northeast. No grave pit was visible and no grave goods. Dating of the skeleton: 4608-4455 calBCE (5699±26 BP, MAMS-47796). Unpublished.

PIE006. P13B483. Chalcolithic. Belongs to the genetic PIE_CA cluster.
Female individual. Settlement mound, bones found in area around an oven. Dating of the skeleton: 4581-4403 calBCE (5666±26 BP, MAMS-47797). Unpublished.

PIE007, PIE016, PIE031. P11N705. Chalcolithic. Belongs to the genetic PIE_CA cluster.
Male individual, 35-45 years old. Grave found west of the tell. Oriented east-to-west, the skeleton was contracted on its left side, facing north. Right hand was positioned on the torso. Grave goods: one bent bronze dagger between legs, skull and other bones from a dog. Dating of the skeleton: 3770-3646 calBCE (4933±25 BP, MAMS-47798)[13].

PIE008. P14T615. Chalcolithic. Belongs to the genetic PIE_CA cluster.

Female individual. Found in the flat settlement, skull facing east. Other bones were found, small hand fragments in front of its face and small rib fragments scattered around. In front of her face lay one lid decorated in Boian style and one bone tool. Dating of the skeleton: 4489-4355 calBCE (5593±26 BP, MAMS-47799)[13].

PIE009. P10J413. Chalcolithic. Belongs to the genetic PIE_CA cluster.

Male individual, >16 years old. Flat settlement. Human bones found in the area between the installation/oven and the clay structure belonging to a burnt house. Second degree relation to PIE010 (P10M644). Dating of the skeleton: 4604-4551 calBCE (5686±29 BP, MAMS-47800). Unpublished.

PIE010. P10M644. Chalcolithic. Belongs to the genetic PIE_CA cluster.

Female individual, 50-60 years old. Grave found in the flat settlement, northeast from the tell. Skeleton in contracted position on its left side, orientation east-to-west. No pit visible, no grave goods. Second degree relation to PIE009 (P10J413). Dating of the skeleton: 4441-4256 calBCE (5485±28 BP, MAMS-47801). Unpublished.

PIE012. P11L273. Chalcolithic. Belongs to the genetic PIE_CA cluster.

Female individual. Mass grave found in the flat settlement, north of the tell. Bones were preserved in good condition. Dating of the skeleton: 4648-4455 calBCE (5705±26 BP, MAMS-47802)[117].

PIE013. P11B023. Chalcolithic. Belongs to the genetic PIE_CA cluster.

Male individual. Settlement mound. Human bones found in a burnt house. Dating of the skeleton: 4536-4362 calBCE (5623±26 BP, MAMS-47803). Unpublished.

PIE014. P10J402. Chalcolithic. Belongs to the genetic PIE_CA cluster.

Female individual, 50-60 years old. Flat settlement north of the tell. Parts from two individuals, badly preserved. Most of the bones were scattered. Dating of the skeleton: 4541-4369 calBCE (5641±27 BP, MAMS-47804). Unpublished.

PIE015. P11L273. Chalcolithic. Belongs to the genetic PIE_CA cluster.

Male individual. Mass grave found in the flat settlement, north of the tell. Bones were preserved in good condition. Dating of the skeleton: 4612-4455 calBCE (5704±26 BP, MAMS-47805)[118].

PIE017. P12B208. Chalcolithic. Belongs to the genetic PIE_CA cluster.

Male individual, juvenile. Settlement mound, from fill between settlement layers. Dating of the skeleton: 4708-4537 calBCE (5761±29 BP, MAMS-47806). Unpublished.

PIE018. P13T213. Chalcolithic. Belongs to the genetic PIE_CA cluster.

Female individual, >50 years old. Flat settlement. Skeleton in contracted position on its left side, east-to-west orientated. Grave was dug into the Neolithic house. No grave goods. Dating of the skeleton: 4494-4357 calBCE (5602±26 BP, MAMS-47807). Unpublished.

PIE019. P10M647. Chalcolithic. Belongs to the genetic PIE_CA cluster.
Male individual, 20-35 years old. Grave found in the flat settlement, north of the tell. Bones were preserved in good condition. Skeleton oriented south-west, contracted on the right side, facing east. No pit visible, no grave goods. Dating of the skeleton: no collagen. Unpublished.

PIE022. P11N707. Chalcolithic. Belongs to the genetic PIE_CA cluster.
Male individual, 20-35 years old. Grave found in the flat settlement, west of the tell. Skeleton oriented north-south, contracted on its right side, facing west. No pit visible, no grave goods. Dating of the skeleton: 4445-4338 calBCE (5531±25 BP, MAMS-47809)[118].

PIE023. P13T210. Chalcolithic. Belongs to the genetic PIE_CA cluster.
Male individual, 6-7 years old. Area with human remains, found in the flat settlement north of the tell. Two children. Skull; other bones could not be found. Dating of the skeleton: 4603-4447 calBCE (5676±29 BP, MAMS-47810). Unpublished.

PIE025. P09F102. Chalcolithic. Belongs to the genetic PIE_CA cluster.
Male individual, >50 years old. Settlement mound. Found in the northern wall of a burnt house. Dating of the skeleton: 4586-4448 calBCE (5678±24 BP, MAMS-47812). Unpublished.

PIE026. P05B123. Chalcolithic. Belongs to the genetic PIE_CA cluster.
Male individual, mature. Settlement mound, area outside the houses. Dating of the skeleton: 4546-4370 calBCE (5651±28 BP, MAMS-47813). Unpublished.

PIE028. P11L280. Chalcolithic. Belongs to the genetic PIE_CA cluster.
Male individual, >50 years old. Flat settlement, skull in east profile. Just the skull was collected, the remaining skeleton was not excavated. Dating of the skeleton: 4493-4356 calBCE (5600±27 BP, MAMS-47814). Unpublished.

PIE029. P11F881. Chalcolithic. Belongs to the genetic PIE_CA cluster.
Male individual. Settlement mound. Outside the houses, among a shell concentration. Dating of the skeleton: 4605-4405 calBCE (4696±26 BP, MAMS-47815). Unpublished.

PIE030. P09J376. Chalcolithic. Belongs to the genetic PIE_CA cluster.
Female individual, 3-4 years old. Flat settlement without clear context. Non-local, according to isotopes. Dating of the skeleton: 4341-4177cal BCE (5419±27 BP, MAMS-47816)[13].

PIE032. P09J390. Chalcolithic. Belongs to the genetic PIE_CA cluster.
Female individual, 20-25 years old. Flat settlement, colluvial deposit. Dating of the skeleton: 4453-4351 calBCE (5574±28 BO, MAMS-47817). Unpublished.

PIE033. P08B114. Chalcolithic. Belongs to the genetic PIE_CA cluster.
Male individual, 16-17 years old. Settlement mound. Calvarium with rounded breaks, found in a dark brownish soil outside the houses. Dating of the skeleton: 4681-4496 calBCE (5734±28 BP, MAMS-47818)[115].

PIE034. P08B108. Chalcolithic. Belongs to the genetic PIE_CA cluster.
Female individual, 15-16 years old. Settlement mound. Cranium with traces of blunt force trauma. Found in a loamy area outside the houses, sandstones. Dating of the skeleton: 4681-4502 calBCE (5741±22 BP, MAMS-47819)[115].

PIE035. P11L273. Individual B. Chalcolithic. Belongs to the genetic PIE_CA cluster.
Female individual, 7.5-9.5 years old. Mass grave found in the flat settlement, north of the tell. Bones were preserved in good condition. Dating of the skeleton: 4607-4457 calBCE (5700±23 BP, MAMS-478020)[115,116].

PIE037. P13L121. Chalcolithic. Belongs to the genetic PIE_CA cluster.
Male individual, >50 years old. Grave in the flat settlement, skeleton oriented east-to-west contracted on its right side, facing south. Oval-shaped pit, no grave goods. Dating of the skeleton: 4346-4249 calBCE (5439±26 BP, MAMS-47821). Unpublished.

PIE038. P12L057. Chalcolithic. Belongs to the genetic PIE_CA cluster.
Female individual, 40-55 years old. Grave in the flat settlement. Skeleton oriented north-south, contracted on its back. No pit visible. Grave goods: fragments from one vessel and half of a spondylus pendant. Dating of the skeleton: 4676-4461 calBCE (5722±26 BP, MAMS-47822). Unpublished.

PIE039. P11N743. Neolithic.
Female individual. Neolithic Pit. Dating of the skeleton: 5319-5132 calBCE (6274±28 BP, MAMS-47823). Unpublished.

PIE041. P10L234. Chalcolithic. Belongs to the genetic PIE_CA cluster.
Female individual, 20-25 years old. Flat settlement north of the tell. Human skull found on shell concentration. Dating of the skeleton: 4611-4457 calBCE (5706±22 BP, MAMS-47824)[117].

PIE042. P13T222. Chalcolithic. Belongs to the genetic PIE_CA cluster.
Male individual, 60-70 years old. Grave found in the flat settlement, north of the tell. Skeleton is oriented east-west, contracted on its right side, facing south. No grave pit, no grave goods. Dating of the skeleton: 4539-4370 calBCE (5638±22 BP, MAMS-47825). Unpublished.

PIE043. P10M640. Chalcolithic. Belongs to the genetic PIE_CA cluster.
Female individual, 35-50 years old. Flat settlement. Single bone in an area between houses. Dating of the skeleton: 4986-4795 calBCE (5996±28 BP, MAMS-47826). Unpublished.

PIE044. P10M645. Chalcolithic. Belongs to the genetic PIE_CA cluster.
Male individual, >20 years old. Flat settlement, area in between houses. Dating of the skeleton: 4533-4357 calBCE (5614±32 BP, MAMS-47827). Unpublished.

PIE048. P11L273. Individual D. Chalcolithic. Belongs to the genetic PIE_CA cluster. Female individual, 20-35 years old. Mass grave found in the flat settlement, north of the tell. Bones were preserved in good condition. Dating of the skeleton: 4678-4493 calBCE (5728±26, MAMS-478028)[118].

PIE050. P12L006. Chalcolithic. Belongs to the genetic PIE_CA cluster.
Male individual, adult. Flat settlement, bones found in a shell concentration. Dating of the skeleton: 4581-4400 calBCE (5664±26 BP, MAMS-47829). Unpublished.

PIE053. P13T214. Chalcolithic. Belongs to the genetic PIE_CA cluster.
Female individual, 20-40 years old. Grave found in the flat settlement, north of the tell. Skeleton in contracted position on its right side, oriented S-N. Some bones are missing. No grave pit is visible and no grave goods were found. Dating of the skeleton: 4492-4356 calBCE (5599±26 BP, MAMS-47830). Unpublished.

PIE054. P18W650. Chalcolithic. Belongs to the genetic PIE_CA cluster.
Male individual, adult. Skeleton found in the western wall of a house in the flat settlement west of the tell. Oriented south-to-north, facing east, with hands placed in front of its face. Second degree relation to PIE058 (P18W642). Dating of the skeleton: 4342-4242 calBCE (5424±27, MAMS-47831). Unpublished.

PIE057. P18W629. Chalcolithic. Belongs to the genetic PIE_CA cluster.
Male individual, 4-5 years old. Skeleton of a child found in the western wall of a house in the flat settlement west of the tell. One small, oval-shaped pit was visible. The skeleton was oriented south-to-north, contracted on its right side. Not all bones were present. Hands were placed in front of its face. After collecting the bones, bones from the basin of skeleton P18W642 appeared under the skull of PIE057 (P18W629). Dating of the skeleton: 4486-4355 calBCE (5589 ±26 BP, MAMS-47832). Unpublished.

PIE058. P18W642. Chalcolithic. Belongs to the genetic PIE_CA cluster.
Male individual, adult. Skeleton found in the western wall of a house in the flat settlement west of the tell. Oriented south-to-north and facing east, hands placed in front of its face. Heavily contracted. Second degree relation to PIE054 (P18W650). Dating of the skeleton: 4341-4242 calBCE (5421±26 BP, MAMS-47833). Unpublished.

PIE060. P18F708. Chalcolithic.

Male individual. Settlement mound. Single bone from a fill layer between two houses. Same individual as P18F738 (PIE061). Dating of the skeleton: 4776-4551 calBCE (5813±25 BP, MAMS-47834). Unpublished.

PIE061. P18F738. Chalcolithic.

Male individual. Settlement mound. Single bone from a fill layer between two houses. Same individual as PIE060 (P18F7089. Dating of the skeleton: 4672-4500 calBCE (5698±34, MAMS-47835). Unpublished.

PIE062. P16H205. Chalcolithic. Belongs to the genetic PIE_CA cluster.

Male individual. Grave. The skeleton is oriented west-to-east, contracted on its right side and facing south. No pit is visible. One small vessel in front of the knees, inside the vessel were found 47 tubular clay and 5 round beads. Dating of the skeleton: 4346-4250 calBCE (5441±26, MAMS-47836)[13].

PIE063. P15T508. Chalcolithic. Belongs to the genetic PIE_CA cluster.

Male individual. Grave in flat settlement. Skeleton is oriented west-to-east, in contracted position, laying on its right side and facing south. No pit visible, fragments from a pot were collected near the bones. Dating of the skeleton: 4549-4371 calBCE (5657±26 BP, MAMS-47837)[13].

PIE064. P15B730. Chalcolithic. Belongs to the genetic PIE_CA cluster.

Female individual. Settlement mound, human bones found in the filling unit. Dating of the skeleton: 4589-4409 calBCE (5672±26 BP, MAMS-47838). Unpublished.

PIE065. P12L006. Chalcolithic. Belongs to the genetic PIE_CA cluster.

Female individual. Flat settlement, bones found in a shell concentration. Dating of the skeleton: 4676-4460 calBCE (5721±27 BP, MAMS-47839). Unpublished.

PIE069. P16B068. Chalcolithic. Belongs to the genetic PIE_CA cluster.

Female individual. Settlement mound, from the profile. Dating of the skeleton: 4588-4451 calBCE (5683±22 BP, MAMS-47840). Unpublished.

PIE074. P17H907. Chalcolithic. Belongs to the genetic PIE_CA cluster.

Female individual. Grave found in the flat settlement east of the tell. Skeleton is oriented northwest-to-southeast, contracted on its left side. No pit visible, no grave goods. Dating of the skeleton: 4360-4256 calBCE (5475±25, MAMS-47842). Unpublished.

PIE075. P17H923. Chalcolithic. Belongs to the genetic PIE_CA cluster.

Female individual. Grave found in the flat settlement east of the tell. Skeleton is oriented northwest-to-southeast, contracted on its left side. No pit visible, no grave goods. Dating of the skeleton: 4444-4270 calBCE (5508±26 BP, MAMS-47843). Unpublished.

PIE076. P16H203. Chalcolithic. Belongs to the genetic PIE_CA cluster.
Male individual. Grave found in the flat settlement east of the tell, a lot of bones are missing. Oriented east-to-west. No pit was visible. Dating of the skeleton: 4345-4249 calBCE (5437±26 BP, MAMS-47844). Unpublished.

PIE077. P16H214. Chalcolithic. Belongs to the genetic PIE_CA cluster.
Female individual. Flat settlement. Fragment of a skull and other bones, badly preserved, oriented north-to-south. No pit grave, no grave goods. Dating of the skeleton: 4339-4176 calBCE (5411±26 BP, MAMS-47845). Unpublished.

PIE078. P16N513. Early Bronze Age
Male individual. Grave disturbed by plowing; fragments from the skull were found southeast of the concentration of bones. No pit grave. Dating of the skeleton: 3335-3026 calBCE (4463±25, MAMS-47846). Unpublished.

3.1.3 Varna I, Bulgaria

N 43.22° E 27.91°

Excavated by the Varna Regional Museum of History

The Copper Age cemetery is situated on the western coast of the Black Sea in the western industrial zone of the current-day Bulgarian harbor city on a slope ~200 m north of the Varna Lake.

The site was discovered by chance in 1972 when a cable trench was dug in an empty area between two factories. It was subsequently excavated on a space of 7500 m^2 from the Regional Historical Museum of Varna under the direction of Ivan Ivanov well into the 1990s[119,120]. In terms of cultural history, the burial site belongs to the Late Chalcolithic Varna group of the Karanovo VI–Gumelniţa–Kodžadermen complex (KGK VI) which extends from the Danube delta to the northern edge of the Rhodope Mountains in the mid- and late 5th millennium BCE. Through archaeologically modeled radiocarbon dates the timespan of burial practices can be confined to approximately 4600-4300 calBCE[58,59]. A small group of only three graves from another cemetery, Varna II, which is located a few kilometers to the west of Varna I, is considered to be the immediate predecessor[121].

The significance of Varna I presents itself in the copious grave goods and its early date. Together with a few exceptional finds in settlements and the grave group of Varna II, it constitutes the earliest presently known burials with gold objects and heavy copper tools in the world. The unequal distribution[59] of the objects among the 329 burials, symbolic graves and depositions at Varna I has given rise to the hypothesis that the Copper Age communities on the Western Black Sea coastline may have been already socially differentiated. Even though Varna I shows this social stratification in grave goods this is not reflected in the genetic ancestry where no genetic outlier individuals were detected.

Human skeletal remains from Varna included in this study:

VAR002. Skeleton 121-I. Belongs to the genetic VAR_CA cluster.
Female individual, 25-30 years old. The skeleton is oriented northeast-to-southwest, contracted on its right side. Oval pit, no grave goods. Dating of the skeleton: 4608-4399 calBCE (5672±34 BP, OxA-13252). Published[58].

VAR003. Skeleton 121_II. Belongs to the genetic VAR_CA cluster.
Female individual, 50-70 years old. Published[60].

VAR004. Skeleton 255. Belongs to the genetic VAR_CA cluster.
Male individual, 18-25 years old. Skeleton is oriented northeast-to-southwest, in an extended position. No pit visible, fragments from a vessel were collected to the right of the bones. Dating of the skeleton: 4686-4496 calBCE (5732±33 BP, OxA-13254). Published[58].

VAR006. Skeleton 92. Belongs to the genetic VAR_CA cluster.
Male individual, ~30 years old. Skeleton is oriented northeast-to-southwest, in an extended position. No pit visible. A group of findings around the head: two clay vessels, three flint blades and a copper awl. To the left of the body: antler implement, two flint tools and a copper ax. Ochre around the face and on the chest. Dating of the skeleton: 4491-4358 calBCE (5600±23 BP, MAMS-51329). Unpublished.

VAR008. Skeleton 173. Belongs to the genetic VAR_CA cluster.
Male individual, >50 years old. Skeleton is oriented northeast-to-southwest, contracted with its face down. No pit visible. Next to the head: a clay vessel. In the area of the waist: a clay vessel and a fragment of a flint tool. Dating of the skeleton: 4703-4541 calBCE (5765±27 BP, MAMS-51330). Unpublished.

VAR009. Skeleton 182. Belongs to the genetic VAR_CA cluster.
Male individual. >50 years old. Skeleton is oriented northeast-to-southwest, in an extended position. No pit visible, fragments from a vessel were collected to the right of the bones. On the

chest: necklace of *Spondylus* beads, two flint blades, stone adze, a copper awl and an antler implement. Dating of the skeleton: 4581-4368 calBCE (5659±31 BP, OxA-23622). Published.

VAR010. Skeleton 37. Belongs to the genetic VAR_CA cluster.
Male individual, 20-25 years old. Skeleton is oriented northeast-to-southwest, in an extended position. No pit visible, fragments from a clay vessel and a flint tool were collected to the right of the bones. In the filling of the pit: two fragments of two *Spondylus* bracelets. Dating of the skeleton: 4688-4503 calBCE (5749±27 BP, MAMS-51323). Unpublished.

VAR011. Skeleton 28. Belongs to the genetic VAR_CA cluster.
Male individual, 30-35 years old. The grave is disturbed, no pit visible. The bones are scattered. They belong to two individuals, but most of them are from this male individual. Fragments of a clay vessel, flint tool and antler implement were found. Dating of the skeleton: 4485-4347 calBCE (5574±31 BP, OxA- 23611). Published[60].

VAR012. Skeleton 197. Belongs to the genetic VAR_CA cluster.
Adult female individual. The grave is disturbed, no pit visible. The bones of an adult person are scattered. Close to them a flint tool was found. Dating of the skeleton: 4619-4450 calBCE (5689±38 BP, OxA-19925). Unpublished.

VAR016. Skeleton 32. Belongs to the genetic VAR_CA cluster.
Male individual, 14-16 years old. Skeleton is oriented northeast-to-southwest, in an extended position. No pit visible, fragments from a clay vessel were collected to the left of the skull. On both sides of the skull: one gold ring (earrings). On the right shoulder: a copper axe. Dating of the skeleton: 4535-4368 calBCE (5631±35 BP, OxA-19870). Published[60].

VAR017. Skeleton 22. Belongs to the genetic VAR_CA cluster.
Female individual, 20-25 years old. The skeleton is oriented northeast-to-southwest, contracted on its right side. No pit visible, fragments from a clay vessel were collected between the bones. Dating of the skeleton: 4672-4458 calBCE (5714±24 BP, MAMS-51324). Unpublished.

VAR018. Skeleton 294. Belongs to the genetic VAR_CA cluster.
Male individual, ~20 years old. Skeleton is oriented northeast-to-southwest, in an extended position. No pit visible. To the right behind the skull: four clay vessels and an antler implement. Necklace of *Spondylus* beads on the neck, flint blade on the left shoulder. Beads of *Spondylus* around the feet. Dating of the skeleton: 4531-4354 calBCE (5608±32 BP, OxA-23626). Published[60].

VAR019. Skeleton 152. Belongs to the genetic VAR_CA cluster.
Male individual, ~35 years old. Skeleton is oriented northeast-to-southwest, in an extended position. No pit visible. On both sides of the skull: clay vessel. Behind the skull: a clay vessel

and an antler implement. On the neck: a necklace of serpentinite and *Spondylus* beads. On the left forearm: a *Spondylus* bracelet. To the left of the skeleton: flint scraper, stone adze and two clay vessels. Dating of the skeleton: 4700-4546 calBCE (5773±24 BP, MAMS-51325). Unpublished.

VAR020. Skeleton 157. Belongs to the genetic VAR_CA cluster.
Adult male individual. The grave is disturbed, no pit visible. The bones of an adult individual are scattered. Between them two *Spondylus* beads, two *Spondylus* bracelets and fragments of a clay vessel were found. Dating of the skeleton: 4603-4452 calBCE (5687±27 BP, MAMS-51331). Unpublished.

VAR021. Skeleton 285. Belongs to the genetic VAR_CA cluster.
Female individual (infans II). The grave is disturbed, no pit visible. The bones of a young individual (*infans II*) are scattered. To the north of them: fragments of three *Spondylus* bracelets. Between the bones: fragments of two clay vessels. Dating of the skeleton: 4547-4408 calBCE (5661±23 BP, MAMS-51332). Unpublished.

VAR022. Skeleton 42. Belongs to the genetic VAR_CA cluster.
Male individual, ~20 years old. Skeleton is oriented northeast-to-southwest, in an extended position. No pit visible. To the right of the skull: two clay vessels. Dating of the skeleton: 4586-4448 calBCE (5677±24 BP, MAMS-51326). Unpublished.

VAR023. Skeleton 127. Belongs to the genetic VAR_CA cluster.
Adult male individual. Same individual as ANI160 from [29]. Skeleton is oriented northeast-to-southwest, in an extended position. No pit visible. Under the skeleton: flint tool. In the filler above the head: stone adze. Dating of the skeleton: 4685-4499 calBCE (5735±31 BP, OxA-24041). Published[60].

VAR026. Skeleton 139a. Belongs to the genetic VAR_CA cluster.
Male individual, 25-30 years old. The grave is disturbed, no pit visible, the bones are scattered. Next to them: fragments of a clay vessel. Dating of the skeleton: 4606-4372 calBCE (5668±33 BP, OxA-23620). Published[60].

VAR027. Skeleton 99. Belongs to the genetic VAR_CA cluster.
Female individual, 30-35 years old. The skeleton is oriented northeast-to-southwest, contracted on its right side. No pit visible. Behind and under the skull: two clay vessels and a stone polisher. On the skull: diadem of *Spondylus* beads. On the neck: necklace of snails' shells. Next to the mouth: a clay vessel. On the right arm: *Spondylus* bracelet. Dating of the skeleton: 4674-4461 calBCE (5721±24 BP, MAMS-51327). Unpublished.

VAR030. Skeleton 194. Belongs to the genetic VAR_CA cluster.

Male individual, 16-19 years old. Skeleton is oriented northeast-to-southwest, in an extended position. No pit visible. Behind the skull: a clay vessel.To the right of the skull: flint tool. On the chest: stone adze. Dating of the skeleton: 4545-4371 calBCE (5650±24 BP, MAMS-51333). Unpublished.

VAR032. Skeleton 155. Belongs to the genetic VAR_CA cluster.
Male individual, ~20 years old. Skeleton is oriented northeast-to-southwest, in an extended position. No pit visible. To the right behind the skull: four clay vessels. Necklace of *Spondylus* and serpentinite beads on the neck. On the waist: stone adze and copper awl. Two *Spondylus* bracelets on the right arm and forearm. Dating of the skeleton: 4547-4408 calBCE (5661±23 BP, MAMS-51328). Unpublished.

VAR033. Skeleton 158. Belongs to the genetic VAR_CA cluster.
Female individual (infans I). The skeleton is oriented northeast-to-southwest, contracted on its right side. No pit visible. Around the skull: three clay vessels. Necklace of *Spondylus*, serpentinite and marble beads on the neck. On the left arm: four *Spondylus* bracelets. In the area of the legs: *Spondylus* and serpentinite beads in two rows. Dating of the skeleton: 4711-4551 calBCE (5787±30 BP, OxA-13688). Published[60].

VAR034. Skeleton 75. Belongs to the genetic VAR_CA cluster.
Female individual, ~30 years old. The grave is disturbed, no pit visible, the bones are scattered. Between them: fragments of clay vessels and gravel. Dating of the skeleton: 4681-4502 calBCE (5740±23, MAMS-51334). Unpublished.

3.1.4 Yunatsite, Bulgaria

N 42.23°, E 24.26°

Excavated by the Institute of Archaeology with the Museum at the Bulgarian Academy of Sciences (IAM-BAS)

Tell Yunatsite, also known as Ploskata Mogila (the Flat Mound), is among the largest tell settlements in Bulgaria with a maximum diameter of 110 x 100 m and a height 12 m above ground. The site is situated in southern Bulgaria, at the western periphery of the Upper Thracian Lowland, 6 km northwest of the town of Pazardzhik and around 1 km southwest of the village of Yunatsite. The tell was established on a low terrace on the right bank of the Topolnitsa River, near its confluence with the Maritsa River. Over time, the bed of both rives shifted. The

Topolnitsa River was subjected to corrections by building dikes in the 20th century. The Maritsa River is nowadays 3 km away from the tell but it was much closer to it during the Chalcolithic. Tell Yunatsite was located in a fertile plain area, surrounded on three sides by mountains: the Rhodope Mountains to the south (~16 km away), the Rila and Ihtimanska Sredna Gora Mountains to the west (~25–30 km away) and the Sashtinska Sredna Gora Mountain to the north, whose southern slopes are ~7–8 km away.

The location of the site represented an excellent strategic point and an exceptional communicative center with the Upper Thracian Lowland connected by suitable routes to the west to the Struma Valley and Sofia Field and from there, through the valleys of the Nishava and Iskar Rivers to the Danube. It is not surprising that the Via Diagonalis, one of the main roads of the Roman Empire, passed the tell about 500 m to the south. The Maritsa River provided an easy connection to the central parts of the Upper Thracian Lowland to the east and thence to the Aegean coast to the south. The Mesta Valley divides the Rila and Rhodope Mountains and can also be used as an alternative route to the north Aegean coast.

This favorable location, providing occupation for various ethnic groups during a period of six thousand years, resulted in the accumulation of deposits and the formation of the tell.

The first test trench made in 1939 already provided evidence that the sequence of the site was extremely important for the prehistory of southeastern Europe[122]. The excavation brought to light a medieval cemetery (13th – 14th c.), a Roman period settlement (2nd – 4th c.), a Thracian period settlement (6th – 5th c. BCE), a thick Bronze Age layer (3rd millennium BCE), as well as a Chalcolithic layer (5th millennium BCE)[123,124]. It is not possible to define precisely in which time period the earliest settlement was established since virgin soil has not yet been reached.

Since 1976 large-scale archaeological excavations have been launched under the direction of R. Katincharov, a researcher at the Institute of Archaeology with the Museum at the Bulgarian Academy of Sciences (IAM–BAS). A Russian (Soviet until 1991) team of the Institute of Archeology at the USSR Academy of Sciences headed by N. Y. Merpert also participated in the excavations between 1982 and 2000[123]. The excavated area was situated in the eastern half of the tell and covered around one third of its entire area. The latest (last) Chalcolithic settlement of the Copper Age (occupation level BI) was reached and excavated on a large scale.

Since 2001 the excavations have continued under the direction of Y. Boyadzhiev (National Archeological Institute with Museum at the Bulgarian Academy of Sciences at NAIM–BAS). A joint Bulgarian-Greek project led by Y. Boyadzhiev and Ioanis Aslanis (the

National Hellenic Research Foundation – Greek and Roman Antiquity) was implemented between 2002 and 2011[124]. The excavations of the residential part of the occupation level BI are completed and date back to the Chalcolithic. Furthermore, trenches at the western and southern periphery of the site were made, and a fortification wall and a ditch, as well as a large deep negative feature at the inner side of the wall, were found and date back to the Chalcolithic. Occupation level BII was unearthed in the northern half of the excavated area and a large settlement beyond the boundaries of the tell was discovered.

Since 2012 the archaeological excavations have been carried out under the direction of Y. Boyadzhiev and K. Boyadzhiev (NAIM-BAS) with the support of the Ministry of Culture of the Republic of Bulgaria (2012–2018), the Balkan Heritage Foundation (2013–2018), the Stone & Compass company (2015–2017), the Municipality of Pazardzhik (2017) and the Pazardzhik Regional Museum of History (2017–2018). The excavations focus on two areas: excavations of Chalcolithic ditch and the deep negative structure at both sides of the fortification wall as well as V. Mikov's trench that started in 1939, and the strip along its southern side. The current research aims to define the sequence of the tll site more precisely by excavating down to the earliest settlement.

Sequence of the tell

LAYER A (top layer):

The thickness of the layer varies from 0.20-0.40 m at the northern half (line E) to 0.80-0.90 m in the central part (lines M-P). It was not possible to define preserved occupation levels. The area was occupied in the early and late Iron Age as well as in the Roman period. A small stone fortification was constructed in the Roman period, and it was probably related to the ancient settlement to the south of the tell[122,125].

Layer B – Chalcolithic:

Layer B is dated back to the Chalcolithic based on the Bulgarian chronology[126] (5th mill. BCE). There was a break in the occupation of the tell between the Late Chalcolithic and the EBA. This gap is evidenced by a hiatus, a layer whose thickness varies as a result of the slope on one hand and the concentrations of burnt Chalcolithic house debris on the other. The people who built up the first Bronze Age settlement leveled up the terrain and as a result, the EBA houses were built immediately on top of the leveled Chalcolithic house debris in some sectors of the central (highest) part of the tell. In contrast, the hiatus layer consisting of loose grayish-black soil with few small ceramic shards is preserved in other sectors. The hiatus is very well documented in the southern periphery of the tell, where its thickness reaches up to 0.40 m. The

available ^{14}C dates yielded by the Late Chalcolithic occupation level reveal that the hiatus between the two periods lasted for around one thousand years[127,128]. This is the time of the so-called Transitional period between the Chalcolithic and the Bronze Age, during which a decrease in the population of the entire territory of present-day Bulgaria is documented.

The latest Chalcolithic settlement (occupation level BI) is the only one dated to this period and, when unearthed, covered the entire excavated area, which spans around one third of the total area of the tell[129]. This occupation level provides remarkable information that is crucial for knowledge about the processes and events leading to the end of the Chalcolithic cultures of the Balkans. It was revealed that the settlement was destroyed as a result of an enemy attack. Ten burnt buildings were unearthed and only one intact house was excavated (№ 8) which is around 12 x 5.40 m large[129]. The rest of the houses remained partially or fully covered by the central cross-section that is the not excavated part of the tell, or were situated in the periphery of the mound, being later destroyed by erosion or by intrusions, such as the EBA ditch and V. Mikov’s trench in 1939. It seems that most of the houses were between 10 m and 13 m long and between 6 m and 8 m wide. These houses functioned for a long period of time, which is evidenced by the numerous layers of floor plasters, whose thicknesses varied between 0.15 m and 0.20 m.

A solid defensive wall enclosing the tell was documented in the latest Chalcolithic occupation level, together with a ditch on the outside of it[130]. However, the strong defensive facilities were not able to save the inhabitants of the latest Chalcolithic settlement. It was conquered, burnt and the residents were slaughtered. Some of the human skulls bear signs of injuries[129,131]. A number of bodies (~50 individuals) were discovered buried under the remains of the houses destroyed by fire and bones of more than 20 individuals were found scattered between the houses and some were found in a crouched position on their side. Most probably, they were hastily buried inhabitants of the village, who had died during the siege before the village was conquered. There was no sign of grave pits or grave goods.

The pottery assemblage of the latest Chalcolithic settlement is typical for the last (3rd) phase of the Chalcolithic. It comprises typical features of the Karanovo VI–Gumelniţa–Kodžadermen complex (KGK VI) and the Krivodol-Sălcuța-Bubani complex[132,133]. The series of ^{14}C dates yielded by this occupation level corresponds to the dates of the late Karanovo VI and Varna cultures, but it is slightly earlier than the dates of the late Krivodol culture[129].

Ongoing excavations in the last few years have provided evidence of continuous occupation throughout the 5th millennium BCE (Early, Middle and Late Chalcolithic), complex social organization, early copper and gold metallurgy.

LAYER Б – Early Bronze Age:

Layer Б is entirely dated back to the EBA according to Bulgarian chronology and terminology[126]. Its thickness varies between 3.5 and 4.5 m in the various sectors of the excavated area. 17 occupation levels were defined, 14 of which yielded a total of 30 ^{14}C dates. The analysis of the samples shows that the EBA settlements existed between 3000 and 2300 cal. BC: Б XVII-XV – EBA I (3000/2950 – 2870/2820 cal. BC); Б XIV-IX – EBA II (2835/2785 – 2640/2590 cal. BC); Б VIII-I – EBA III (2605/2555 – 2350/2300 cal. BC)[124, 126-128].

The earliest Bronze Age settlers resettled the tell after a break in occupation of around one thousand years. They found a mound accumulated during the Chalcolithic, rising at least 6 m above the surrounding area. It was situated in an extremely favorable location and provided a naturally protected living place. It has been suggested that the settlers were few in number because the earliest EBA houses (occupation level BHVII) were situated only along the periphery of the tell. A palisade surrounding the northern half of the tell at the inner side of the settlement was constructed together with the houses. It seems that the population increased rapidly because houses were built on the entire excavated area during the occupation level БXV. There was a ditch and a rampart on the northern side of the palisade[126,128,135], and a wooden bridge above the ditch in front of the palisade entrance. The remains were documented in occupation level БXIV.

The number of the inhabitants continued to increase and the settlement of the occupation level БXIII-XII (EBA II) extended beyond the boundaries of the tell, reaching 100-150 m to west, covering an area of ca. 30.000-40.000 m^2. The deposits of the EBA II and EBA III occupation levels of the satellite settlement are around 1.70 m thick[52]. Bronze Age deposits were also documented ~70 m to the south of the tell (unpublished information from the 2019 archaeological campaign), and three occupation levels were defined. A defensive wall made from solid wooden posts and intertwined with sticks and plastered clay was erected along the western periphery of the tell in the same period.

Similarly, to other EBA sites, infant jar burials were discovered under the floors of some houses[136] (Extended Data Fig. 1h). They were found in occupation levels XVII-X during the first half of the occupation on the tell. Such burials are not documented in later settlements. An infant jar burial was also excavated in 2019. The burial pit was cut into the floor of the latest occupation level of the satellite EBA settlement documented in trench 20 which is situated to the south of the tell. Two adult graves were also found close to each other inside of the tell and were attributed to the first EBA settlement (level XVII).

Human skeletal remains from Yunatsite included in this study:

Chalcolithic burials inside the buildings:

YUN003. Skeleton 69. Chalcolithic. Belongs to the genetic YUN_CA cluster. Adult female individual, 25-35 years old. Found under the burnt destructions of building 5a.

YUN007. Skeleton 87. Chalcolithic. Belongs to the genetic YUN_CA cluster. Adult male individual, 40-50 years old. Found under the burnt destructions of building 3. Dating of the skeleton: 4451-4346 calBCE (5551±27 BP, MAMS-45480).

YUN012. Skeleton 23. Chalcolithic. Belongs to the genetic YUN_CA cluster. Female individual, 2-3 years old. Found under the burnt destructions of building 1.

YUN013. Skeleton 77/81. Chalcolithic. Belongs to the genetic YUN_CA cluster. Adult male individual, 30-35 years old. Found under the burnt destructions of building 12. Dating of the skeleton: 4517-4367 calBCE (5624±26 BP, MAMS-45483).

YUN014. Skeleton 67. Chalcolithic. Belongs to the genetic YUN_CA cluster. Adult female individual, 45-55 years old. Found under the burnt destructions of building 11. Dating of the skeleton: 4459-4354 calBCE (5577±29 BP, MAMS-45484).

YUN015. Skeleton 24. Chalcolithic. Belongs to the genetic YUN_CA cluster. Adult male individual, 30-39 years old. Found under the burnt destructions of building 1. Dating of the skeleton: 4450-4342 calBCE (5541±28 BP, MAMS-45485).

YUN019. Skeleton 69A/80. Chalcolithic. Belongs to the genetic YUN_CA cluster. Juvenile, feminine individual, Juvenilis. Found under the burnt destruction of building 5.

YUN021. Skeleton 92. Chalcolithic. Belongs to the genetic YUN_CA cluster. Female individual, 3-5 years old. Found under the burnt destructions of building 5. Dating of the skeleton: 4341-4247 calBCE (5431±25 BP, MAMS-45486).

YUN025. Skeleton 56A. Chalcolithic. Belongs to the genetic YUN_CA cluster. Female individual, 1-2. Found under the burnt destructions of building 6.

YUN031. Skeleton 126. identical twins/same individual as YUN006. Skeleton 127.

YUN049. Skeleton 69. identical twins/same individual as YUN003.

Chalcolithic burials outside of the buildings:

YUN005. Skeleton 104. Chalcolithic. Belongs to the genetic YUN_CA cluster. Adult female individual, 50-60 years old. Heavily destroyed by an EBA pit. Dating of the skeleton: 4453-4356 calBCE (5570±24 BP, MAMS-45478).

YUN009. Skeleton 102. Chalcolithic. Belongs to the genetic YUN_CA cluster.

Male individual, 3-5 years old. Supine position. Located to the north of building 6. Dating of the skeleton: 4450-4346 calBCE (5548±28 BP, MAMS-45482).

YUN026. Skeleton 78. Chalcolithic. Belongs to the genetic YUN_CA cluster.
Adult male individual, 35-40 years old. Flexed position, lying half on the back, half on the right side. W–E orientation. Found in the space between buildings 2 and 3. Dating of the skeleton: 4537-4373 calBCE (5637±26 BP, MAMS-45489).

YUN027. Skeleton 83. Chalcolithic. Belongs to the genetic YUN_CA cluster.
Adult female individual, 25-35 years old. Flexed position on the right side. E-W orientation. Located to the north of building 3. Dating of the skeleton: 4528-4371 calBCE (5632±24 BP, MAMS-28134).

YUN028. Skeleton 98. Chalcolithic. Belongs to the genetic YUN_CA cluster.
Adult female individual, 60-70 years old. Disturbed, head pointing to the WSW. Dating of the skeleton: 4517-4368 calBCe (5625±26 BP, MAMS-45490).

YUN048. Skeleton 103. Chalcolithic. Belongs to the genetic YUN_CA cluster.
Adult male individual, 20-30 years old. Heavily destroyed by an EBA pit. Dating of the skeleton: 4545-4449 calBCe (5657±24 BP, MAMS-45503).

Chalcolithic burial outside of the tell:

YUN001. Burial 110. Chalcolithic. Belongs to the genetic YUN_CA cluster.
Female individual. Buried in supine position. E-W orientation. Located just outside of the tell, in its southern periphery. Dating of the skeleton: 4505-4360 calBCE (5614±31 BP, MAMS-45476).

Skeletal remains from the “Big negative feature”:

YUN006. Skeleton 127. Chalcolithic. Belongs to the genetic YUN_CA cluster.
Female individual. Petrous bone right. Fallen from level BI. Dating of the skeleton: 4462-4356 calBCE (5589±26 BP, MAMS-45479).

Skeletal remains of 12 early Bronze Age individuals:

YUN033. Burial 45. Early Bronze Age. Belongs to the genetic YUN_EBA cluster.
Female individual, 6 months old. Baby urn-burial. Level Б XVI/XVII, building 52. Dating of the skeleton: 3323-2926 calBCE (4428±27 BP, MAMS-45491).

YUN034. Burial 11. Early Bronze Age. Belongs to the genetic YUN_EBA cluster.

Female individual, 6-9 months old. Baby urn-burial. Level Б XIII, building 25. Dating of the skeleton: 2908-2759 calBCE (4239±26 BP, MAMS-45492).

YUN035 + **YUN036**. Burial 4. Early Bronze Age. Belong to the genetic YUN_CA cluster.

Female individuals, 6 months old. Baby urn-burial. Level Б X, outside a building, sq. E9. Dating of the skeleton: 3323-2926 calBCE (4167±25 BP, MAMS-45493).

YUN037 + **YUN038**. Burial 8. Early Bronze Age. Belong to the genetic YUN-EBA cluster.

Male individuals, 3-6 months old. Baby urn-burial. Level Б XII, building 14. Dating of the skeleton: 2911-2765 calBCE (4248±24 BP, MAMS-45494).

YUN039. Burial 3. Early Bronze Age. Belongs to the genetic YUN_EBA cluster.

Female individual, 6 months old. Baby urn-burial. Level Б XI, building 12A. Dating of the skeleton: 2898-2700 calBCE (4213±24 BP, MAMS-45495).

YUN041. Burial 10. Early Bronze Age. A genetic outlier individual.

Female individual, 6 months old. Baby urn-burial. Level Б XIII, building 20. Dating of the skeleton: 2878-2638 calBCE (4161±27 BP, MAMS-45496).

YUN042. Burial 45C. Early Bronze Age. Belongs to the genetic YUN_EBA cluster.

Female individual, 6 months. Baby urn-burial. Dating of the skeleton: 2885-2675 calBCE (4181±25 BP, MAMS-45497).

YUN043. Burial 6. Early Bronze Age. Belongs to the genetic YUN_EBA cluster.

Female individual. Baby urn-burial. Level Б XI, 4,8 m south of building 11. Dating of the skeleton. 2879-2636 calBCE (4162±26 BP, MAMS-45498).

YUN045. Burial 22. Early Bronze Age. Belongs to the genetic YUN_EBA cluster.

Female individual, 9-12 months old. Baby urn-burial. Level Б XV, building 32. Dating of the skeleton: 2913-2879 calBCE (4270±24 BP, MAMS-45500).

3.1.5 Boyanovo Mounds, Yambol District, Bulgaria

N 42.28°, E 26.63°

Excavation by the Yambol Regional Historical Museum

The Boyanovo funerary complex, comprising mounds 8007, 7004, and 6009, is located 2.7 km east of the Tundzha River and 2 km north of Boyanovo village in the Yambol province

of southeastern Bulgaria[136]. This area is at the southern edge of the Thracian Plain where east-to-west running ridge-lines rise from the plain some 50-100 m above the Tundzha River. The enclosed valleys saw agricultural communities proliferate from the Late Neolithic, while the ridge lines attracted mound builders from the early Bronze Age on. Environmentally, the area falls into the forest-steppe, bordered by the steppe to the northeast, forests and montane forests to the west, and Pontic coastal environments to the south and east.

The cluster of mounds at Boyanovo was excavated in 2010 in a rescue campaign preceding the planned expansion of a nearby limestone quarry[137,138]. Mound 8007 sat directly atop a limestone outcrop (~235 masl) that dominated the local landscape and contained some 20 burials, which yielded six of the seven aDNA samples from this site in the present study, of which 4 belong to the early Bronze Age. One sample came from a burial in mound 6009 which lays some 300 m southeast of 8007. Mound 8007 was the largest of the three mounds, 45 m in diameter and 4.8 m in height. Mound 6009 was the smallest of the cluster of three, measuring 23 m in diameter and 1.6 m high. Mound 7004 yielded no burials. No ^{14}C testing was conducted on the excavated remains due to the rescue nature of the operation, and hence all of the dates in the original reports are cultural and relative.

Excavations confirmed that mound 8007 was constructed and first used for burials in the Early Bronze Age, and was used again during the Late Bronze Age. Later burials from the post-Roman or Medieval era were also found in the surface layers of the mound. In total, the remains of 20 burials containing 23 individuals were uncovered within the mound. Twelve burials at the core of the mound were dated to the early Bronze Age. One burial (#13) contained Bronze Age ceramic and a silver pendant while another (#7) contained a ceramic jug dating to EBA III (neither yielded viable DNA). Other burials were dated to the EBA because of their location near or below #13 at the very center of the mound in a rock cut grave (19, 20, and 21) or in nearby stone-lined pits (#13 and 17) and crouched position on side or back, covered by ochre (#18, 12, 9, and 7). Some crouched burials did not contain any ochre (#2 and 8) and are differentiated from other crouched burials (#4,5,6,10, and 15) only by darker grey-brown soil. As two of the latter burials (#5 and 15) contained LBA ceramics, the dark-soil layer was interpreted as an LBA event. Given the only differentiating feature among burials #2, 8, 4, 6, and 10, however, is the colour of soil (all share the crouched position), the date remains tenuous. Two supine, extended burials (#3, 14) were discovered near the surface of the mound and provisionally dated on the basis of the location and supine position to the post-Roman period. None of these late burials had any grave goods associated with them so the specific period is difficult to determine. Four burials were excavated in mound 6009, one post-Roman near the

surface and three inside a stone structure at the base of the mound. The similarity of the internal stone construction to the central grave in 8007 as well as two associated handmade vessels date the burials #2-4 in 6009 to the Bronze Age.

The location, chronology, and burial context of the early human remains recovered from the mortuary site of Boyanovo link these individuals with contemporary pastoral trends. In a previous study, stable isotope analysis of collagen extracted from the bones of 14 individuals was applied to investigate dietary patterns within the Boyanovo population. The results indicated that humans interred at Boyanovo relied upon terrestrial fauna for their dietary protein. Carbon stable isotope values of the population reflect a mixed C3-C4 diet, either from regular, direct consumption of C4 plants (especially millet) or the frequent consumption of animals grazed or foddered on a high-C4 diet[139].

Human skeletal remains from Boyanovo included in this study:

BOY001. Burial mound 8007, #2. Belongs to the genetic BOY_EBA cluster.
Crouched position of an adult male individual (25-30 years old). Soil in the grave was dark grey-brown. Dating of the skeleton: 2895-2680 calBCE (4205±24 BP, MAMS-47224)

BOY008. Burial mound 8007, #9. Belongs to the genetic BOY_EBA cluster.
Crouched position of an adult male individual (60-65 years old), covered with ochre. Dating of the skeleton: 2899-2697 calBCE (4213±24 BP, MAMS-47227)

BOY009. Burial mound 8007, #12. Belongs to the genetic BOY_EBA cluster.
Crouched position of an adult male individual (60-70 years old), covered with ochre. Dating of the skeleton: 2901-2697 calBCE (4218±25 BP, MAMS-47228)

BOY014. Burial mound 8007, #18. Belongs to the genetic BOY_EBA cluster.
Crouched position of a female individual (25-30 years old), covered with ochre. Dating of the skeleton: 3316-2923 calbCE (4421±25 BP, MAMS-47229)

BOY019. Burial mound 6009, #4. A genetic outlier individual.
Dating of the skeleton: 2898-2697 calBCE (4212±24 BP, MAMS-47230)

3.2 **Northwestern Black Sea Region**

3.2.1 Orlivka-Kartal, Reni District, Odesa Region, Ukraine

N 45.32°, E 28.44°

Excavation by the Odesa Archaeological Museum.

The multicultural site of Orlovka-Kartal represents an extensive and structurally diversified archaeological complex consisting of settlements, special production zones, tumuli, flat cemeteries and defensive structures[140]. The site occupies a vast area extended along an east-to-west line for about 3.5 kilometers. The eastern border of the complex is marked by a Roman rampart and a ditch running from the southeast to the northwest and crossing the eastern periphery of the modern-day village of Orlivka. Almost all of the village is part of the archaeological site which is evidenced by numerous archaeological finds which are regularly found within the village.

The main archaeological objects are concentrated in the western part of the site, about 1.5 km west of the village. They are situated on a narrow promontory, extending from the east to the west and bordering the Cahul Lake to the north and surrounded by the Danube to the south. The western extremity of the promontory is occupied by a multilayer settlement which consists of two parts: the western part is located on top of an elevation with the name of Kamennaya Gora (English: Stone Hill) which represents a kind of fortified acropolis. Initially this elevation covered an area of about 3-4 ha, but now it is almost completely destroyed due to schist mining. A lower settlement or a suburb is attached to the acropolis from the east. Cultural deposits of the settlement belong to different time periods and are about 3 m thick.

The deepest layer of the deposits is attributed to a settlement of the Gumelniţa culture (the Bolgrad variant), and is covered by the late Eneolithic layer represented by the Cernavodă I culture. Upper layers include materials of the early Iron Age, the Greek period of the 3rd-4th centuries BCE, and the late Roman period. Sporadic finds of the Middle Ages are documented as well. All these cultural layers are uncovered both in the acropolis and in the lower settlement. The settlement of the Cernavodă I culture was protected by seven defensive ditches up to 3 m deep. According to radiocarbon dates the settlement of the Gumelniţa culture can be dated to the middle of the 5th millennium BCE whereas the settlement of the Cernavodă I culture is dated to the second and third quarters of the 4th millennium BCE.

An extensive multicultural cemetery is located approximately 200 meters east of the settlement. Several hundred uncovered graves are related to the early Iron Age. Thirty-eight graves can be attributed to the Cernavodă I culture of the late Eneolithic. Additionally, isolated burials of the Yamnaya and Babino cultures, the late Bronze Age and the Sarmatian culture have been found in the cemetery.

The late Eneolithic graves are combined in small groups or situated separately at a great distance from one another. Only in the central part of the cemetery, graves arranged in two rows and extended in a meridian direction can be detected. Burial structures are represented by rectangular or oval pits frequently with irregular shape. Three monoliths around graves were uncovered, although there are no signs of the burial mounds. Every grave followed the burial rite of inhumation. Three main types of positions of the deceased are documented. The first is a crouched position on the back with the arms extended along the body. The second type is characterized by individuals being buried in a crouched position on their back with the body tilted to the left or right side. In this case one arm was extended along the body and the other arm was half-bent with the hand at or on the pelvis. The third type shows individuals laying in a crouched position on their left side with different dispositions of the arms. The individuals are mainly oriented with the skulls towards east or southeast. Grave offerings are scarce in all three types of burials, and graves often only contain one vessel, isolated shards, flints or beads. Only one grave contained two vessels.

Cernavodă I-associated human skeletal remains included in this study:

KTL001. Grave 10. Belongs to the KTL A group.

The grave was uncovered in square A'/7, at a depth of 3.09-3.11 m, in the layer of the Cernavodă I culture within the settlement. It was partially covered by grave 9. The young male was in a crouched position on his right side with his skull to the southeast. His left arm was stretched to the knees. His right arm was bent and the forearm stretched forth. Dating of the skeleton: 3502-3343 calBCE (4609±26 BP, MAMS-48810).

KTL002. Grave 10. Belongs to the genetic KTL_B cluster.

The irregular oval pit (1.5×1.1 m, 1.55 m deep) in the cemetery was extended along a northeast–southwest line. The female individual (30-35 years old) was in a crouched position on her left side with her skull to the northeast. Her left arm was bent and with the forearm stretched forth. Her right arm was also bent with her hand on the left forearm. Dark-brown remains of an organic mat was found on the bottom of the pit. A bowl with an S-shaped profile made of shell-tempered clay was placed at the parietal part of the skull. A small ceramic fragment was placed in front of the individual. A lump of ochre laid at the left shoulder. Dating of the skeleton: 3641-3528 calBCE (4806±25 BP, MAMS-48811).

KTL003. Grave 14. Belongs to the KTL A group.

The oval burial pit (1.85×1.8 m, 1.75 m deep) was extended along an east-to-west line. The northern side of the pit was destroyed by a later structure. The female individual (25-30 years

old) was buried in a crouched position on her left side with the skull to the southeast. The left arm was destroyed by a later pit. The right arm was bent in the elbow with the forearm in the area of the abdominal cavity, perpendicular to the body. The bones were covered with red ochre. The burial chamber was filled by a layer of wattle and daub lumps. The center of the layer had a funnel-shaped hole which had been closed by a clay plug. Fragments of thin-walled shell-tempered pottery were present in the filling of the pit. Grave goods were absent. Dating of the skeleton: 3696-3530 calBCE (4840±26 BP, MAMS-48812).

KTL004. Grave 20. Belongs to the genetic KTL_B cluster.

The burial pit had an approximately rectangular shape with strongly rounded corners (1.67×1.17 m on the upper level and 1.4×1.0 m on the bottom level, 1.41-1.38 m deep). It was extended along a southeast-to-northwest line. Narrow ledges of 15 cm height were present along the perimeter of the pit except on the eastern side. The skeleton of an adult woman laid in a crouched position on the left side with the skull to the southeast. The arms were bent with the hands in front of her face. Dark-brown remains of an organic mat covered the bottom of the pit. Grave goods consisted of an amphora and a milk jug made of shell-tempered clay. Dating of the skeleton: 3952-3792 calBCE (5064±27 BP, MAMS-48813).

KTL005. Grave 36. Belongs to the genetic KTL_B cluster.

The burial structure and the skeleton of an adult male individual were severely disturbed by later intrusions. The burial structure was represented by remains of an oval pit 1.4-1.45 m deep that extended along a southeast-to-northwest line. The individual was in a crouched position on the left side with the skull to the southeast. The left arm was slightly bent and with the forearm stretched to the knees. The right arm was also bent and laid with the forearm on the chest perpendicular to the body. The bottom of the pit was covered by dark-brown remains of a plant mat. Grave goods were absent. Dating of the skeleton: 3763-3638 calBCE (4905±28 BP, MAMS-48814).

KTL006. Grave 168. Belongs to the KTL A group.

The northern part of the burial pit and the lower part of an adult male skeleton were destroyed by a later structure. According to preserved parts, the pit likely had an oval shape with an orientation along a southeast-to-northwest line. The pit is narrower towards the bottom through ledges cut out in the walls. Dimensions of the pit in the upper part could be 1.8×1.5 m, 1.5×1.0 m in the bottom part. The bottom of the pit went down from the northwest to the southeast to 15 cm. It was on the depth of 0.98-1.12 m from the modern surface. The individual laid in a crouched position on his back with the skull to the southeast. The left arm was bent and with the forearm drawn aside from the body. Only the humerus of the right arm is preserved and also

extended aside from the body. The inventory included flint flakes which were placed under the head of the individual. Dating of the skeleton: 4156-3959 calBCE (5205±29 BP, MAMS-48815).

KTL007. Grave 193. Belongs to the KTL A group.

The grave was situated in the center of a cromlech. The cromlech was built of flat stones (dark-green schist) which were laid flatways in several layers and represented a semicircle open from the southern side. The base of the cromlech was 0.75-0.8 m deep compared to the modern surface. The masonry was especially massive in the northeastern sector where its length was 60-100 cm and width reached 40-45 cm made of tightly packed medium sized (20×25 cm, 30×15 m, etc.) and large sized stones (50×30 cm, 60×25 cm, etc.). The second semicircle was situated at the outer side of the inner semicircle. It consisted of small flat stones which were laid in a broken manner in one layer on the depth of 0.6-0.7 m. The presence of two semicircles can be considered as a constructive element of the structure. The inner semicircle had an external diameter of 7 m, the outer semicircle of 8 m. The cromlech was disturbed in some places by intrusions of the early Iron Age, however, in the southern part such intrusions were absent, so the gap in this sector seems to have been left intentionally.

The rectangular pit (1.7×1.2 m, 1.7 m deep) was extended along an east-to-west line with some insignificant deviations. The adult female individual was buried in a crouched position on her back with the head to the east. The body was slightly tilted to the left side. The left arm was bent and with the forearm stretched ahead. The right humerus laid along the body whereas bones of the forearm were absent. The bones of the skeleton were covered with red ochre. Two small lumps of ochre were situated right next to the skull. Two fragments of Eneolithic pottery were placed at the left shoulder and left of the pelvis. The bottom of the pit was covered with remains of an organic mat. Dating of the skeleton: 3971-3801 calBCE (5104±27 BP, MAMS-48816).

KTL008. Grave 204.

The rectangular burial pit with rounded corners in its western part (1.7×1.05-0.85 m, 1.3 m deep) was extended along an east-west axis. A low ledge (15-25 cm wide and 5 cm high) was made along the eastern side of the pit. Remains of two individuals were found in the grave. Both skeletons were severely disturbed by animal holes. The position of an adolescent individual in the southern part of the pit could not be determined due to the bad preservation of the skeleton. The second skeleton of an adult female was in a crouched position on the back with her head to the south-east. Dark-brown remains of a plant mat were traced under the second

skeleton. The grave inventory consisted of tubular, cylindrical and disk-shaped beads and shells. Dating of the second skeleton: 3756-3636 (4895±26 BP, MAMS-48817).

3.2.2 Mayaki, Belyaevka District, Odesa Region, Ukraine

N 46.23°, E 30.16°

Excavation by the Odesa Archaeological Museum

This archaeological complex of the Usatove culture consists of a settlement and a cemetery located on the southern edge of the village of Mayaki. The complex occupies the promontory of the fourth river terrace rising above the valley of the left bank of the Dniester River. The settlement is almost completely destroyed by the erosion process. The system of joined deep ditches was studied in a preserved part of this area. The cemetery is situated about 200 m northwest of the settlement and includes both tumulus and flat burials[141]. Flat burials are situated separately or constitute isolated clusters consisting of two to four graves and are designated as separate complexes. It was suggested that flat graves were initially covered with low mounds, which later eroded or were leveled by plowing. The cemetery was excavated in 1974, 1975 and 1986. In total, 45 graves were uncovered which contained remains of 55 individuals. Later, two more individual graves were studied. Additionally, graves of the early Eneolithic, the Yamnaya culture, the late Bronze Age, the Scythian and the Sarmatian cultures were found in the area of the cemetery. Samples for DNA analysis were obtained from one tumulus and three flat complexes of the Usatove culture, one grave of the Yamnaya culture and one grave of the early Bronze Age

Eneolithic human skeletal remains from Majaky included in this study:

Tumulus 3 is 0.3 m high with a diameter of 10 m containing 8 graves and one cult pit of the Usatove culture. One individual from grave 12 produced genome-wide data.

MAJ020. Grave 12 (Usatove culture). Belongs to the genetic MAJ cluster.
The oval burial pit with a niche of 0.6 m height and a ledge of 0.4 m height (1.3×0.75 m on the upper level and 1.3×1.15 m on the bottom, 1.4 m deep) was extended along a northeast-to southwest line. The female individual (50-60 years old) was buried in a crouched position on the left side with her head to the northeast. Her left arm was bent with the hand in front of the

face. The right arm was bent with the hand on the elbow of the left arm. Strips of brown ochre were traced on the skull. Patches of brown rot were traced on the bones of the limbs. Grave goods consisted of two vessels made of shell-tempered clay. Dating of the skeleton: 3948-3794 calBCE (5058±21 BP, MAMS-48825).

Complex II consisted of a grave of the Usatove culture and a grave of the Sarmatian culture. The individual from the Usatove culture produced genome-wide data.

MAJ002. Grave 1 (Usatove culture). Belongs to the genetic MAJ cluster.
The oval burial pit (1.6×1.1 m, 0.9 m deep) was extended along a northeast-to-southwest line. The adolescent female individual was buried in a crouched position on the left side with the head to the northeast. The arms were bent with the hands in front of her face. Remains of brown dye were traced on the calvarium. A vessel made of shell-tempered clay was in the left hand of the individual (not preserved). Dating of the skeleton: 4441-4261 calBCE (5498±26 BP, MAMS-48818).

Complex III consisted of four graves of the Usatove culture and one destroyed grave. Two individuals produced genome-wide data (for the second see further down).

MAJ008. Grave 14 (Usatove culture). Belongs to the genetic MAJ cluster.
The trapezoid pit (1.5×1.2-0.9 m, 1.0 m deep) was extended along an east-to-west line. The female individual (25-30 years old) was buried in a crouched position on the left side with her skull to the east. The arms were bent with the hands in front of the face. Strips of reddish-brown ochre were evidenced on the frontal bone and the facial bones of the skull. Grave goods consisted of one painted bowl and two vessels made of shell-tempered clay. Dating of the skeleton: 4231-3989 calBCE (5270±28 BP, MAMS-48821).

Complex IV contained two graves of the Usatove culture. One individual produced genome-wide data.

MAJ023. Grave 13. belongs to the genetic MAJ cluster.
The trapezoid burial pit (2×1.5-1.25 m, 1.15 m deep) with a ledge of 0.2 m height and 0.4 m width was extended along a southeast-to-northwest line. The female individual (30-35 years old) was buried in a crouched position on the left side with the head to the southeast. Her arms were bent with the hands in front of her face. Traces of brown ochre are preserved on the skull. Grave offerings included a bronze dagger, a painted amphora with a lid and three vessels made of shell-tempered clay. Dating of the skeleton: 4239-4050 calBCE (5311±25, MAMS-48826).

Complex V consisted of two graves of the Usatove culture. One individual produced genome-wide data.

MAJ003. Grave 12. Belongs to the genetic MAJ cluster.

The trapezoid pit (1.4×1.1 m on the upper level and 1.4×1.3 m on the bottom level, 0.85 m deep) was extended along a northeast-to-southwest line. The male child (7-10 years old) laid in a crouched position on the left side with the skull to the northeast. His arms were bent with the hands in front of his face. Patches of rot were traced on the bones of the limbs. The inventory included a copper awl, a vessel made of shell-tempered clay, two anthropomorphic figurines and four jet beads. Dating of the skeleton: 3900-3653 calBCE (4978±28 BP, MAMS-48819).

Complex VII contained one grave of the Usatove culture. The individual from this grave produced genome-wide data.

MAJ009. Grave 16. Belongs to the genetic MAJ cluster.

The rectangular burial pit (1.4×0.9 m, 0.7 m deep) was extended along a northeast-to-southwest line. The male child (7-12 years old) was buried in a crouched position on the left side with the skull to the northeast. Strips of brown ochre were observable on the lower jaw. Patches of rot were traced on the skull and the bones of the legs. Grave goods consisted of three vessels made of shell-tempered clay. Dating of the skeleton: 4445-4345 calBCE (5545±27 BP, MAMS-48822).

Tumulus 1 is 1.0 meter high with a diameter of 35-50 meter containing 22 graves of different chronological periods. The primary mound was built above grave 9 of the Usatove culture. Later, graves of the Yamnaya culture, the late Bronze Age and later periods were added into the mound. One individual of the Yamnaya culture and one individual of the early Bronze Age produced genome-wide data.

Human skeletal remains from Early Bronze Age burials included in this study:

MAJ019. Grave 18 (Yamnaya culture). Belongs to the genetic MAJ_EBA cluster.

The rectangular burial pit (1.2×0.9 m, 1.4 m deep) was extended from the southwest to the northeast. The adult female individual was buried in a crouched position on her back with the head to the southwest. The arms were bent with the hands on the chest. Traces of ochre were observable on the skull. Grave goods consisted of two vessels. Dating of the skeleton: 2887-2669 calBCE (4185±26 BP, MAMS-48824).

MAJ017. Grave 22 (Early Bronze Age). Belongs to the genetic MAJ-EBA cluster.

The oval burial pit (1.4×0.9 m, 1.0 m deep) was extended along the southeast-to-northwest line. The adult male individual was buried in a crouched position with the head to the west. Grave goods were absent. Dating of the skeleton: 2910-2705 calBCE (4242±27 BP, MAMS-48823).

Complex III consisted of four graves of the Usatove culture and one destroyed grave. Two individuals produced genome-wide data.

MAJ004. Grave 5 (Usatove culture). Belongs to the genetic MAJ_EBA cluster. The oval burial pit (1.9×1.45 m, 0.7 m deep) was extended along a northeast-to-southwest line. There were remains of two individuals in the grave. The first female individual (25-30 years old) was buried in the center of the grave in a crouched position on her left side with the head to the northeast. The left arm was extended along the body. The right arm was bent and laid with the forearm on the chest. The left cheekbone was painted with dark-red ochre. This individual was not part of the genetic analysis. The second female individual (25-30 years old) was buried at the southern side of the pit, left of the first female individual, in a crouched position with the head to the northeast. The arms were stretched to the knees. Strips and patches of red, dark-red, orange and crimson ochre are preserved on the calvarium and facial part of the skull. The grave goods consisted of two vessels made of shell-tempered clay and a microlithic trapezium. Dating of the skeleton: 2863-2508 calBCE (4107±25 BP, MAMS-48820).

3.2.3 Usatove-Bolshoy Kuyalnik, Belyaevka District, Odesa Region, Ukraine

N 46.32°, E 30.40°

Excavation by the Odesa Archaeological Museum.

The archaeological site of Usatove-Bolshoy Kuyalnik represents an extensive complex of the Usatove culture which occupies about 20 ha[142]. The complex is located on the edge of a high (about 55 m) steppe plateau of the eastern (right) shore of the Khadzhibey estuary. It is situated on the territory of the villages of Usatove and Bolshoy Kuylanik and consists of a settlement, three tumulus cemeteries and a flat cemetery. The excavations of the complex started in 1921 and with interruptions were continued until 1985. Fifteen tumuli were excavated at the first tumulus cemetery, ten tumuli at the second tumulus cemetery and the flat cemetery were studied completely. The third tumulus cemetery situated northeast of the flat cemetery was completely destroyed. Only a few stone anthropomorphic steles and ceramic fragments were found. Genome-wide data were obtained for some individuals from the tumulus cemetery 2 and the flat cemetery.

The tumulus cemetery 2 was situated on the territory of a modern graveyard and consisted of 10 burial mounds. All tumuli except N 4 are excavated. Genome-wide data were obtained from individuals buried in tumuli 6, 8 and 9.

USV005. Tumulus 6, grave 4. Belongs to the genetic USV cluster.

Information about this tumulus is absent. One female individual produced genome-wide data. Dating of the skeleton: 3646-3529 calBCE (4819±26 BP, MAMS-48809).

Tumulus 9 was 0.25 m high with a diameter of 8-10 m and contained 3 graves and 2 cult pits. One individual produced genome-wide data.

USV004. Grave 3 (Grave 4 in Supplementary Table A). Belongs to the genetic USV cluster.

The grave is represented by a rectangular pit with rounded corners (1.2×1.1 m, 0.82 m deep) and extended along a northeast-to-southwest line. The grave contained remains of three individuals. Bones of an infant painted with crimson ochre were laid at the southwestern corner. Disarticulated bones of an adult male and an adult female were concentrated at the northwestern corner. Grave offerings included a bone bead and a vessel made of shell-tempered clay. The female adult individual was sampled for ancient DNA analyses. Dating of the female skeleton: 3646-3529 calBCE (4819±26 BP, MAMS-48808).

The flat cemetery consisted of five isolated groups of graves and cult pits situated at some distance from one another. Samples for DNA analyses were obtained from the individuals in groups 2, 3 and 5.

Group 2 was situated in the central part of the flat cemetery and consisted of 12 graves and 4 cult pits. One individual from two-level graves 1-2 produced genome-wide data (grave 1, two individuals were buried in grave 1-2).

UBK006. Graves 1-2 (Skeleton 4 in Supplementary Table A). Belongs to the genetic USV cluster.

The grave represented a rectangular pit (1.7×1.3 m, 0.6 m deep) that extended along an east-to-west line and was covered with two stone slabs. A vessel from shell-tempered clay was found under one of the slabs. Fragments of the skull and the limbs of the male child laid at the northern side of the pit (grave 1). Grave offerings consisted of 3 vessels made of shell-tempered clay. Scattered bones of a second skeleton were found at the bottom of the pit. Fragments of the skull were in the eastern part of the pit and bones of the legs were found in the western part of the pit. The deceased probably laid in a crouched position with the head to the east. Grave goods consisted of five vessels made of shell-tempered clay. Dating of the skeleton: 3701-3535 calBCE (4853±23 BP, MAMS-48806).

Group 3 was situated in the central part of the flat cemetery and consisted of 4 graves and 7 cult pits. One individual from grave 4 produced genome-wide data.

UBK003. Grave 4. Belongs to the genetic USV cluster.

The grave represented a partially destroyed rectangular pit (1.3×1.0 m, 0.15 m deep). The adult female individual was buried in a crouched position on the left side with her head to the northeast. Two fragments of a vessel made of shell-tempered clay are positioned near the skeleton. Dating of the skeleton: 3972-3804 calBCE (5110±24 BP, MAMS-48804).

4. Testing for steppe-related ancestry in published CTC-associated individuals

In 2020 Immel and colleagues[46] presented genomic data from individuals associated with the Cucuteni Trypillia Culture (CTC)[11]. One of the main claims of this study was an early contact event between early farmers and pastoralist groups from the steppe regions. However, we question and challenge the interpretation of the results of the *f*-statistics and qpAdm analyses as presented in this study. In particular, the results of the qpAdm ancestry modelling were reported as feasible, but they can hardly be described as well-fit since the standard error for one or more components in the tested models was larger than the components itself and it should therefore be disregarded as a well-fit model (see Supplementary Table 3 in Immel *et al*. 2020). As the claims of this study are directly relevant to our study, we re-analysed the data that was made available to us upon request (data from 3 out of 4 individuals). We repeated the f_4-statistics f_4(*test,* Moldova_CTC; Kalmykia_Yamanya.SG, Mbuti) from the original study but we accounted for possible 'capture' bias by also using shotgun sequence data of Turkey_N[3,144] instead of 1240k SNP capture data as *test* (Supplementary Table O). As a result, we find indeed significant negative values ($|Z|\geq 3$) for Gordinesti and Pocrovca3 (Supplementary Table O), which would be suggestive of gene flow from population C to population B in a test of this form. However, previous ancient DNA studies have shown that the ancestry of Yamnaya-associated individuals is primarily a mixture of two deeper ancestries, which comprise EHG- and CHG[4]-like ancestries. Important for the point in case, Immel *et al*. 2020 did not explore whether any other type of deeper HG ancestry could have led to the excess affinity to steppe-relate ancestry in the reported CTC-associated individuals, in particular whether there was a differential attraction to the basal components of 'steppe ancestry', that is EHG or CHG, or both. In fact, excess affinity to both would be indicative for the actual contribution of 'steppe ancestry'. Therefore, we tested specifically for shared drift between the three Moldova_CTC individuals and CHG and EHG-related ancestry conditioned on early European farmer ancestry with the test f_4(*test*, Moldova_CTC; HG, Mbuti). Here, also accounting for a possible bias by using Turkey N.SG as '*test'* and CHG and Russia_Sidelkino_HG.SG as HG groups. Importantly, Gordinesti and Pocrovca3 do show significant negative values ($|Z|\geq 3$), implying gene flow, but only for Russia_Sidelkino_HG.SG (Supplementary Table O) and not for CHG. To integrate the data from Immel *et al.* 2020 in the test regime we use for the newly reported Ukraine Eneolithic individuals we used f_4(*test*, Moldova_CTC; Yamnaya Samara, Mbuti) with *'test'* being Turkey_N and Germany_EN_LBK (as in Immel *et al*. 2020) and f_4(Turkey_N, Moldova_CTC; HG, Mbuti) with HG being EHG and CHG. The first test results in no

significant f_4-statistics for any of the individuals whereas the second test returns again significantly negative result ($|Z| \geq 3$) only for EHG-related ancestry (Supplementary Table O). Hence, the affinity of Gordinesti and Pocrovca3 to Yamnaya Samara and Kalmykia Yamnaya.SG is likely driven by the attraction to EHG-ancestry, solely, and we conclude that Gordinesti and Pocrovca3 carry no 'steppe-related' ancestry in the true sense of the original definition. By contrast, an analogous test with Ukraine Eneolithic groups used as population B, returns significantly negative f_4-statistics ($|Z| \geq 3$) for both EHG and CHG (this study: Supplementary Table N).

To corroborate these findings, we also tested whether Ukraine Trypillia (Mathieson *et al.* 2018[29]) and Moldovoa_CTC (Immel *et al.* 2020) are symmetrically (equidistantly) related to *test* groups (Ukraine N, EHG, CHG, WHG, Yamnaya Samara) using f_4(Ukraine Trypillia, Moldovo_CTC; *test*, Mbuti). Here, none of the tests deviates significantly from zero ($|Z| \leq 3$) (Supplementary Table O). We therefore included Moldova_CTC in the Ukraine Trypillia individuals reported by Mathieson *et al.* 2018 and use this group in tests which explore the potential contribution of European Neolithic farmer-related sources in this study.

Lastly, we compared the newly reported Ukraine Eneolitic individuals to Moldova_CTC with respect to *test* (Steppe Eneolithic and Yamnaya Samara) using f_4(Moldova_CTC, Ukraine Eneolithic; *test*, Mbuti). Here, Ukraine Eneolithic individuals result in significant negative values ($|Z| \geq 3$) for both *test* populations (Supplementary Table O). This shows that Moldova_CTC and Ukraine Eneolithic are not symmetrically related and that the latter received additional gene flow from Steppe Eneolithic groups who also contributed substantially to subsequent Yamnaya groups.

5. Rationale for selecting source populations

Neolithic groups from Anatolia started to expand into Europe around 7000-6000 BCE. The Neolithic lifestyle was sustainable, and allowed a growth in population size, soon spreading across many regions that allowed for farming. With this movement, autochthonous hunter-gatherers (HG), present during this time, were gradually pushed back further into regions where farming was not yet established or unsuitable, forming mosaics of smaller refugia where they could maintain a hunter-gatherer lifestyle. As a consequence, the genetic profile that came with the early farmers into Europe became the predominant ancestry in most parts of Europe. Since there was initially little to no admixture between local hunter-gatherer groups and expanding Anatolian Neolithic farmers, this early farmer-associated ancestry is still prevailing at the beginning of the Copper Age, approximately 1000-2000 years after the first farmers arrived. However, some Neolithic groups do indeed carry a small proportion of hunter-gatherer-related ancestry due to contact and interaction with hunter-gatherers. Given that all early farmers expanded from smaller groups, and thus are genetically from a similar stock, it is challenging to genetically differentiate early farmer groups. In fact, most early farming groups are best differentiated by the variable amounts and differences sources of HG ancestry.

Here we describe our rationale to select suitable proxies from a large group of early farmer groups for the newly reported individuals PIE039 and the site-based SEE Copper Age groups without simply merging all available Neolithic and Chalcolithic reference groups. We first performed f_4-statistics of the form f_4(*test*, PIE039/SEE CA; HGs, Mbuti) (Extended Data Fig. S3), where '*test*' includes published early to the late Neolithic groups. Since the Neolithic populations only differ by small variations in their HG ancestry, we used a less strict significance cut-off of $|Z|\leq1$ and one standard error to exclude minor differences in genetic ancestry and to restrict our search only to those Neolithic groups that are symmetrical related to PIE039 and the CA groups in an unbiased approach. The respective Neolithic groups were then merged if necessary (Supplementary Table E). In the case of PIE_CA, no Neolithic group was cladal with respect to every HG group tested. Therefore, we followed a tempo-chronological rationale and grouped the two temporally and spatially closest groups, Croatia_N_Cardial and Romania_EN, that were either cladal for some HGs or yielded a consistently low Z-score over all HG comparison (Supplementary Table D). PIE060 yielded significantly negative ($|Z|\geq1$) f_4-statistics for almost all Neolithic groups when compared to WHG, EHG. Therefore, we chose and grouped SEE_N, a group made up of geographically close sites (Yabalkovo N, Krepost N, Dzhulyunitsa N, MalakPreslavents N, Ohoden N,

Romania_EN and LepenskiVir N; see Supplementary Table E). The grouped Neolithic cohorts, as well as single groups, were then used as proxies for ancestry sources in further analyses. In the case of YUN_CA, Greece_N, Turkey_N and Dzhulyunitsa N were grouped as a local reference cohort. However, when this cohort was used as a single source in qpAdm, all models were rejected. Therefore, we also applied the followed a tempo-chronological rationale and chose Dzhulyunitsa N as the closest Neolithic group in time and space, which resulted in a well-fit model for YUN_CA with Dzhulyunitsa N as a single source.

The outlier PIE060 was tested for additional HG ancestry in an f_4-test of the form f_4(SEE_N, PIE060; HGs, Mbuti) to determine a HG proxy (see main text and Supplementary Table F).

6. Rationale for plotting and grouping of distal and proximal source population in qpAdm

For better readability of Fig. 3 in the main text we decided to reduce the number of well-fit models to show but all well-fit models are marked in 'bold' in their respective Supplementary Table. We only excluded well-fit models from adding them to the plot when the result and interpretation of two different well-fit models are the same (see distal qpAdm modelling with EHG or WSHG).

For proximal qpAdm modelling of PIE039 and SEE_CA we used the grouped cohorts mentioned in section 5 and as shown in Supplementary Table E.

For Ukraine Eneolithic groups we tested whether we can distinguish between Russia_Steppe_Maykop, Russia_Steppe_Eneolithic and Russia_Khvalysnk_Eneolithic as source populations for proximal qpAdm modelling. The f_4-test shown in Supplementary Table M results in a grouping of Russia_Steppe_Eneolithic and Russia_Khvalynsk_Eneolithic into 'Steppe Eneolithic' (Supplementary Table E) and an exclusion of Russia_Steppe_Maykop as a possible source population in further analyses.

After selecting putative sources for distal and proximal models for all time periods with the parameter setting 'allSNPs: NO' we then used 'allSNPs: YES' to increase the number of SNPs being used. Due to an increase in informative data for the program, the p-values drop, at times, significantly compared to the 'allSNPs: NO' option, but we find that the ancestry components and the respective proportions for each chosen model vary only slightly in the decimal range. Models calculated with 'allSNPs: NO' were deemed infeasible when the ancestry component of one or more of the tested sources returned a negative estimate.

The comment column in each Supplementary Table tab lists the components which could be removed in a more complex model based on the nested p-value (nested p-value > 0.05) and therefore adds transparency to the model selection and the decision-making process for the models that were shown as best approximations in Fig. 3 of the main text.

7. Intra- and inter-site relatedness using READ

READ was run with the default parameters, and the background-relatedness was estimated using the median value, across all sites. We analysed the sites in groups of temporal groupings. We found that in all cases, that the median P_0 value per site was not significantly different (Supplementary Fig. S1, the blue shaded region) from the between-site median (P_0=0.25), except in the case of PTK which was slightly lower (P_0=0.236). However, all individuals at PTK were classified as “unrelated”, and renormalising their pairwise READ values using a lower median would only make them appear “more unrelated”, yielding no qualitative change in findings (Supplementary Fig. S1).

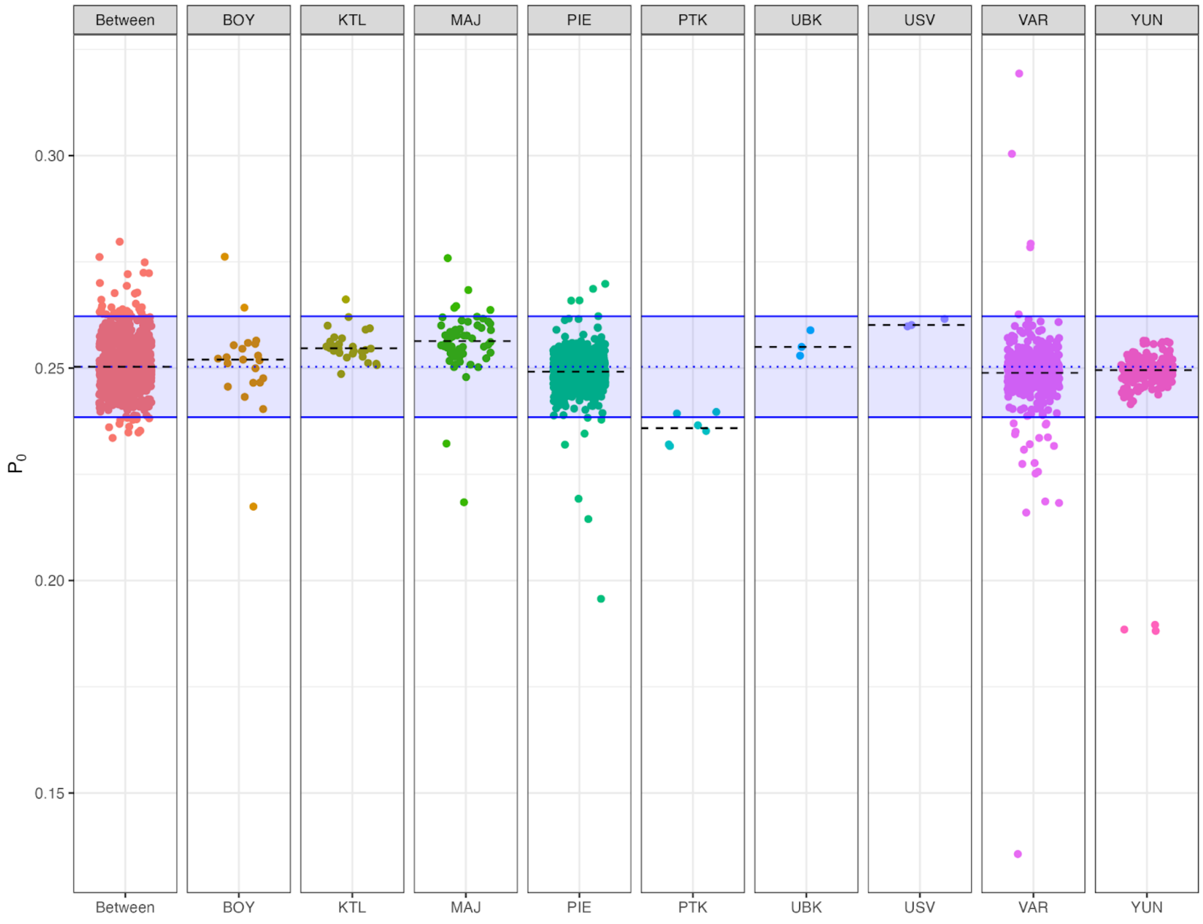

Supplementary Fig. S1. Inter- and intrasite comparison of background relatedness. The blue shaded region shows the median P_0 value between and within sites. Per site medians do not differ from between site median.

References

95. Benecke, N *et al*. Pietrele in the Lower Danube region: integrating archaeological, faunal and environmental investigations. *Documenta Praehistorica* **40,** 175 (2013).

96. Krauß, R., Zäuner, S. & Pernicka, E. in *Metalle der Macht – Frühes Gold und Silber. Metals of Power – Early gold and silver (2/2)* (eds. Meller, H., Risch, R. & Pernicka, E.) 371–387 (Landesamt für Denkmalpflege und Archaologie Sachsen-Anhalt – Landesmuseum für Vorgeschichte Halle (Saale), 2014).

97. Hansen, S. in *Appropriating Innovations: Entangled Knowledge in Eurasia 5000-1500 BCE* (ed. Stockhammer Joseph Maran, P.) 136–148 (Oxbow Books, 2017).

98. Hansen, S. The 4th millennium: a watershed in European Prehistory. *Western Anatolia before Troy. Proto-Urbanisation in the 4th Millenium BC* 243–259 (2014).

99. Subbotin, L. V. *Pamiatniki kultury Gumelnitsa Iugo-Zapada Ukrainy*. Naukova dumka (1983).

100. Râmbu, N. L., Mâtcu M. A., Secară V. M. in *Geografia Moldavskoy SSR*. Lumina, Kishinev (1989).

101. Topchiev, O. G. *Geografia Odeshchiny. Priroda, naselennya, gospodarstvo*. Astroprint (1998).

102. Dergachev, V. A. & Larina, O. V. Pamiatniki kultury Krish Moldovy (s katalogom). *Chişinău: Tipografia Centrala* (2015).

103. Larina, O. V. & Dergachev, V. A. Pamiatniki kultury lineyno-lentochnoy keramiki Respubliki Moldova (svod istochnikov). *Chişinău: Akademiya nauk Moldovy, Institut kulturnogo* (2017).

104. Пиструил. Стоянка Катаржино 1 и проблема идентификации памятников эпохи неолита в степях Северо-Западного Причерноморья. *Stratum plus. Археология и культурная антропология* (2011).

105. Govedarica, B. in *Das Karpatenbecken und die osteuropäische Steppe* (eds. B. Hänsel, J. M.) (Leidorf, Rahden/Westfalen, 1998).

106. Manzura, I. Kultury stepnogo eneolita. *Drevniye kultury Severo-Zapadnogo Prichernomoria* (2013).

107. Morgunova, N. L. Eneolit Volzhsko-Uralskogo mezhdurechya. Orenburg: Izd. *Orenburg. Gos. Ped. Univ* (2011).

108. Todorova, H. Die Protobronzezeit auf der Balkanhalbinsel. *Anatolica* (1993).

109. Manzura, I. Cernavoda I culture. *Nikolova L. The Balkans in Later Prehistory. BAR* (1999).

110. Manzura, I. V. Tokens of time: on anthropological periodization of the Cucuteni-Tripolye culture. *Godišnjak Centra za balkanološka ispitivanja* 23–40 (2019).

111. Petrenko, V. G. Usatovskaya kultura. in *Drevnie kultury Severo-Zapadnogo Prichernomorya* (ed. Bruyako I. V., S. T. L.) (SMIL, 2013).

112. Manzura, I. North-Pontic steppes at the end of the 4th millennium BC: the epoch of broken borders. *Man, culture and society from the Copper Age until the Early Iron Age in Northern Eurasia. Contributions in honour of the 60th anniversary of Eugen Sava* (2016).

113. Chohadzhiev, A. To raise a tell and raise it well: some odd regularities of the early Chalcolithic construction techniques and the building strategies at Tell Petko Karavelovo. *Studia Praehistorica* **15**, (2021).

114. Chohadzhiev, A. & Atanassova, N. Neobichaen grupov grob ot halkolitnata selishtna mogila pri s. Petko Karavelovo. Predvaritelni rezultati. *Izvestiya na Regionalen Istoricheski muzey – Veliko Tarnovo* **34**, 9–58 (2019).

115. Hansen, S., Toderas, M., Reingruber, A., Nowacki, D. & Norgaard, H. Die kupferzeitliche Siedlung Pietrele an der Unteren Donau: Bericht über die Ausgrabungen und geomorphologischen Untersuchungen im Sommer 2010. *Eurasia antiqua: Zeitschrift für Archäologie Eurasiens* **17**, 45–120 (2011).

116. Hansen, S., Toderaş, M. & Wunderlich, J. Pietrele, München: Neolithische und kupferzeitliche Siedlung am See 5200–4250 v. Chr. *e-Forschungsberichte* (2015).

117. Hansen, S., Toderas, M. & Reingruber, A. Pietrele an der Unteren Donau: Bericht über die Ausgrabungen und geomorphologischen Untersuchungen im Sommer 2011. *Eurasia antiqua: Zeitschrift für Archäologie Eurasiens* **18,** 1-68. (2012).

118. Hansen, S., Toderas, M., Reingruber, A., Gatsov, I. & Kay, M. Pietrele," Magura Gorgana": Bericht über die Ausgrabungen und geomorphologischen Untersuchungen im Sommer 2009. *Eurasia antiqua: Zeitschrift für Archäologie Eurasiens* **16**, 43–96 (2010).

119. Ivanov, I. & Lichardus, J. Der Bestattungsritus in der chalkolithischen Nekropole von Varna (mit einem Katalog der wichtigsten Gräber). *Die Kupferzeit als historische Epoche* 125-150 (1991).

120. Slavchev, V. The Varna Eneolithic cemetery in the context of the Late Copper Age in the East Balkans. in *The Lost World of Old Europe. The Danube Valley, 5000-3500 BC* (ed. Anthony, D. W.) 193–210 (Princeton, 2010).

121. Иванов, И. С. *Съкровищата на Варненския халколитен некропол*. (Септември, 1978).

122. Миков. Селищна могила при с. Юнаците (Пазарджишко). *Годишник на Пловдивската народна библиотека и* (1940).

123. Mazanova. Erforschungsgeschichte der Tellsiedlungen Yunatzite, Pasardzikregion. *Boyadzhiev & Terzijska-Ignatova* (2011).

124. Boyadzhiev, Y. *et al.* Yunatsite: Ein Bulgarisch-Griechisches Grabungsprojekt. Die Jahre 2002-2008. *The Golden Fifth Millennium. Thrace and Its Neighbour Areas in the Chalcolithic. Proceedings of the International Symposium in Pazardzhik, Yundola* 21-37 (2011).

125. Катинчаров, Мерперт, Титов & Мацанова. Селищна могила при село Юнаците, Пазарджишко. *Том I. София* (1995).

126. Boyadzhiev, Y. Chronology of Prehistoric Cultures in Bulgaria. in *Prehistoric Bulgaria* (eds. Bailey, D. W. & Panayotov, I.) vol. 22 149–191 (Prehistoric Press, 1995).

127. Юнаците. Эпоха бронзы. *T. II, 1 [= Tell Yunatsite, Bronze Age], Moscow* (2007).

128. Boyadzhiev, Y. Tell Yunatsite: development and absolute chronology of the settlements from the beginning of the Chalcolithic to the Early Bronze Age. *Neolithic and Copper Age between the Carpathians and the Aegean Sea: Chronologies and Technologies from the 6th to 4th Millennium BCE* (2015): 381-393.

129. Matsanova, V. & Mishina, T. The Latest Late Chalcolithic Settlement at Tell Yunatsite: Plan and Architectural Remains. *Communities in Transition: The Circum-Aegean Area in the 5th and 4th Millennia BC, Oxford, 293,* 314 (2018).

130. Boyadziev, Y. in *The town in the Bulgarian lands (according to archaeological data). Reports from the National scientific conference dedicated to the life and activity of Vera Antonova – one of the notable scholars in Medieval Bulgarian studies* (ed. Georgiev, P.) 53–65 (2014).

131. Бужилова, А. П. in *Homo sapiens: История болезни.* (2005).

132. Todorova, H. & Matsanova, V. Late Chalkolithic Ceramic Style at Yunatsite Tell (Approach to the Systematization of the Ceramics from the Newly Excavated Levels). *BAR INTERNATIONAL SERIES* (2000).

133. Todorova, H. The Ornamentation of Late Chalcolithic Pottery from Yunatsite Tell, Pazardzhik District (Systematization and Analysis). *BAR INTERNATIONAL SERIES* (2003).

134. Mazanova, V. Die Struktur der Siedlungen im Siedlungshügel Junazite während der Frühbronzezeit. *Early Bronze Age settlement patterns in the Balkans* (1996).

135. Бояджиев, Я., Бояджиев, К. & Петров, В. Селищна могила Юнаците, Пазарджишко. in *Археологически открития и разкопки през 2019 г.* 325–329 (2020).

136. Bakărdžiev, S., Iliev, I. Kurgane der frühen bis späten Bronzezeit im Bezirk Jambol, Südostbulgarien (eds. S., Echt, R., Heyd, V., & Preda-Bălănică, B.), Archaeolingua (2020).

137. Bakardzhiev, S., c & Rusev, Y. Spasitelni arheologicheski prouchvanya na mogilen nekropol v m.“Baylar Kayryak”, zemlishte na s. Boyanovo, oblastYambol. *Arheologicheski otkritya i razkopki prez 2010 g* (2011).

138. Ross, S. A., Nekhrizov, G. & Tzvetkova, J. The Tundzha Regional Archaeology Project: Surface Survey, Palaeoecology, and Associated Studies in Central and Southeast Bulgaria, 2009-2015 Final Report. *The Tundzha Regional* (2018).

139. Privat, K., Sobotkova, A., Bakardzhiev, S. & Russeva, V. Excavation and Palaeodietary Analysis of Human Remains from Boyanovo, Bulgaria. *Excavation and Paleodietary Analysis* 325–341 (2017).

140. Govedarica, B. & Manzura, I. The Copper Age Settlement of Kartal in Orlovka (Southwest Ukraine). *Neolithic and Copper Age between the Carpathians and the Aegean Sea. Chronologies and Technologies from the 6th to the 4th Millennium BCE. International Workshop Budapest*, 437-457 (2012).

141. Patokova, E. F., Petrenko, V. G., Burdo, N. B. & Polishchuk, L. Usatovskiy mogilnik Mayaki. in *Pamyatniki tripolskoy kultury v Severo-Zapadnom Prichernomorie.* (Naukova Dumka, 1989).

142. Patokova, E. F. Usatovskoe poselenie i mogilniki. in (Naukova Dumka, 1979).

143. Kılınç, G. M. *et al.* The Demographic Development of the First Farmers in Anatolia. *Curr. Biol.* **26**, 2659–2666 (2016).
