# Supplementary material for: Early contact between late farming and pastoralist societies in southeastern Europe
Source: Nature. 2023 Jul 19;620(7973):358–65. doi: 10.1038/s41586-023-06334-8 (PMC10412445; doi:10.1038/s41586-023-06334-8)
Supplement: Supplementary file 2 — Reporting Summary [file 41586_2023_6334_MOESM2_ESM.pdf]

nature portfolio

Corresponding author(s): Sandra Penske
Wolfgang Haak

Last updated by author(s): Jun 1, 2023

Reporting Summary

Nature Portfolio wishes to improve the reproducibility of the work that we publish. This form provides structure for consistency and transparency in reporting. For further information on Nature Portfolio policies, see our Editorial Policies and the Editorial Policy Checklist.

Statistics

For all statistical analyses, confirm that the following items are present in the figure legend, table legend, main text, or Methods section.

n/a	Confirmed	
☐	☒	The exact sample size (*n*) for each experimental group/condition, given as a discrete number and unit of measurement
☐	☒	A statement on whether measurements were taken from distinct samples or whether the same sample was measured repeatedly
☐	☒	The statistical test(s) used AND whether they are one- or two-sided<br>*Only common tests should be described solely by name; describe more complex techniques in the Methods section.*
☒	☐	A description of all covariates tested
☐	☒	A description of any assumptions or corrections, such as tests of normality and adjustment for multiple comparisons
☐	☒	A full description of the statistical parameters including central tendency (e.g. means) or other basic estimates (e.g. regression coefficient) AND variation (e.g. standard deviation) or associated estimates of uncertainty (e.g. confidence intervals)
☐	☒	For null hypothesis testing, the test statistic (e.g. *F*, *t*, *r*) with confidence intervals, effect sizes, degrees of freedom and *P* value noted<br>*Give P values as exact values whenever suitable.*
☒	☐	For Bayesian analysis, information on the choice of priors and Markov chain Monte Carlo settings
☒	☐	For hierarchical and complex designs, identification of the appropriate level for tests and full reporting of outcomes
☒	☐	Estimates of effect sizes (e.g. Cohen's *d*, Pearson's *r*), indicating how they were calculated

Our web collection on statistics for biologists contains articles on many of the points above.

Software and code

Policy information about availability of computer code

Data collection	No specific software was used for data collection. All software used for the processing of raw sequencing data and generation of genotype files is listed below.
Data analysis	The following freely available software was used for data analyses. The corresponding citations are provided in the Material & Methods section: EAGER (1.92.56), AdapterRemoval (v2.3.0), BWA (v 0.7.12), DeDup (v 0.12.1), MapDamage (v 2.0.9), samtools (v 1.3), pileupCaller (v 1.4.0.2), bamUtils (v 1.0.13), ANGSD (0.910), contamMix (v1.0 12), ADMIXTOOLS (5.1) (qp3Pop, qpDstats, qpWave, qpAdm), EIGENSOFT package (v 7.2.1), smartpca (v 16000), Haplogrep 2 (v2.4.0), R (v 3.6.2), DATES (v 753), ancIBD (0.4), hapROH (0.64), GLIMPSE (1.0.1), OxCal (v4.4.2), READ (no versioning), Geneious (v 2019.2.3), HOPS (v 0.2), MALT (v.0.4.0)

For manuscripts utilizing custom algorithms or software that are central to the research but not yet described in published literature, software must be made available to editors and reviewers. We strongly encourage code deposition in a community repository (e.g. GitHub). See the Nature Portfolio guidelines for submitting code & software for further information.

Data

Policy information about availability of data

All manuscripts must include a data availability statement. This statement should provide the following information, where applicable:

- Accession codes, unique identifiers, or web links for publicly available datasets
- A description of any restrictions on data availability
- For clinical datasets or third party data, please ensure that the statement adheres to our policy

Genomic sequence data (BAM format) is available at the European Nucleotide Archive under project accession number PRJEB62503. The published genotype data compiled and annotated as Allen Ancient DNA Resource (AADR v44.3) and that was used for comparative analyses is available here: https://reich.hms.harvard.edu/allen-ancient-dna-resource-aadr-downloadable-genotypes-present-day-and-ancient-dna-data.
The human mitochondrial revised Cambridge Reference Sequence (NC 012920.1): https://www.ncbi.nlm.nih.gov/nuccore/251831106.
The human reference genome GrCh38 (hg38): https://www.ncbi.nlm.nih.gov/datasets/genome/GCF_000001405.26/
The human reference genome GrCh37 (hg19): https://www.ncbi.nlm.nih.gov/datasets/genome/GCF_000001405.13/
The 1000 human genomes reference panel: http://hgdownload.cse.ucsc.edu/gbdb/hg19/1000Genomes/phase3/

Human research participants

Policy information about studies involving human research participants and Sex and Gender in Research.

Reporting on sex and gender	not applicable
Population characteristics	not applicable
Recruitment	not applicable
Ethics oversight	not applicable

Note that full information on the approval of the study protocol must also be provided in the manuscript.

Field-specific reporting

Please select the one below that is the best fit for your research. If you are not sure, read the appropriate sections before making your selection.

☒ Life sciences ☐ Behavioural & social sciences ☐ Ecological, evolutionary & environmental sciences

For a reference copy of the document with all sections, see nature.com/documents/nr-reporting-summary-flat.pdf

Life sciences study design

All studies must disclose on these points even when the disclosure is negative.

Sample size	No statistical methods were used to determine ancient DNA sample size a priori. Sample sizes for ancient groups/population depends entirely on availability and preservation of human skeletal remains associated to archaeologically described cultures or techno-complexes.
Data exclusions	We processed and screened samples from 216 prehistoric individuals, of which 135 were deemed suitable for downstream analyses, following pre-defined data quality and authentication criteria, described in the Methods section. Data from specimens that showed insufficient levels of ancient DNA content or high levels of DNA contamination were excluded from further analyses.
Replication	We study unique entities of past populations and did not use different treatments or variations of data analyses, so replication is not applicable. We recognize that individuals from the same region and time period of the past show similarities, and that their particular ancestry composition does not exist in the same form anymore today. Genome-wide data with hundreds of thousands of SNPs allows for multiple realisations of the sample history.
Randomization	Individuals are grouped by time period (archaeological culture, radiocarbon date), geographic region and genetic similarity. Randomisation is thus not relevant to this study.
Blinding	As the archaeological and anthropological context of our samples (date and location, etc.) is critical to the interpretation of the data, blinding is not applicable to our study.

Reporting for specific materials, systems and methods

We require information from authors about some types of materials, experimental systems and methods used in many studies. Here, indicate whether each material, system or method listed is relevant to your study. If you are not sure if a list item applies to your research, read the appropriate section before selecting a response.

Materials & experimental systems

n/a	Involved in the study
☒	☐ Antibodies
☒	☐ Eukaryotic cell lines
☐	☒ Palaeontology and archaeology
☒	☐ Animals and other organisms
☒	☐ Clinical data
☒	☐ Dual use research of concern

Methods

n/a	Involved in the study
☒	☐ ChIP-seq
☒	☐ Flow cytometry
☒	☐ MRI-based neuroimaging

Palaeontology and Archaeology

Specimen provenance	Specimen come from excavations supported by the Regional Museum of History - Veliko Tarnovo, the German Archaeological Institute - Berlin (Germany), the Institute for Archaeology "Vasile Pârvan" of the Romanian Academy of Sciences - Bucharest (Romania), the Institute for Geography of the Goethe University - Frankfurt/Main (Germany), the Varna Regional Museum of History - Varna (Bulgaria), the National Institute of Archaeology with the Museum at the Bulgarian Academy of Sciences (IAM-BAS) - Sofia (Bulgaria), the Regional Museum of History - Pazardzhik (Bulgaria), the Yambol Regional Historical Museum - Yambol (Bulgaria), and the Odesa Archaeological Museum - Odesa (Ukraine), with primary contact persons provided in Supplementary Table A.<br>All specimens were collected and analyzed with permissions from the respective local organisations for the handling of the archaeological material, and represented by local curators and collaboration partners who are listed among the co-authors of this study.
Specimen deposition	Specimens are stored at the Regional Museum of History - Veliko Tarnovo, the German Archaeological Institute - Berlin (Germany), the Institute for Archaeology "Vasile Pârvan" of the Romanian Academy of Sciences - Bucharest (Romania), the Institute for Geography of the Goethe University - Frankfurt/Main (Germany), the Varna Regional Museum of History - Varna (Bulgaria), the National Institute of Archaeology with the Museum at the Bulgarian Academy of Sciences (IAM-BAS) - Sofia (Bulgaria), the Regional Museum of History - Pazardzhik (Bulgaria), the Yambol Regional Historical Museum - Yambol (Bulgaria), and the Odesa Archaeological Museum - Odesa (Ukraine).<br>Specimens will be returned to the respective heritage organization and museums after completion of the joint collaborations. DNA extract and libraries will remain stored at the ancient DNA laboratories of the Max Planck Institute for Evolutionary Anthropology, Jena & Leipizg, Germany.
Dating methods	New AMS 14C dates were obtained from ultra-filtrated collagen. Collagen extraction and 14C measurements were carried out at the Curt-Engelhorn-Zentrum Archäometrie gGmbH, Mannheim, Germany.

☒ Tick this box to confirm that the raw and calibrated dates are available in the paper or in Supplementary Information.

Ethics oversight	No ethics oversight was required.<br>The archaeological and anthropological researchers included as coauthors of this study were granted permissions to sample skeletal elements for the purpose of ancient DNA analyses. All steps in the analyses followed standard ethical guidelines with regards to respectful handling, documentation, storage, transport, sampling and processing of human skeletal elements, with support from the Regional Museum of History - Veliko Tarnovo, the German Archaeological Institute - Berlin (Germany), the Institute for Archaeology "Vasile Pârvan" of the Romanian Academy of Sciences - Bucharest (Romania), the Institute for Geography of the Goethe University - Frankfurt/Main (Germany), the Varna Regional Museum of History - Varna (Bulgaria), the National Institute of Archaeology with the Museum at the Bulgarian Academy of Sciences (IAM-BAS) - Sofia (Bulgaria), the Regional Museum of History - Pazardzhik (Bulgaria), the Yambol Regional Historical Museum - Yambol (Bulgaria), the Odesa Archaeological Museum - Odesa (Ukraine) who approved and provided guidance on the study protocol.

Note that full information on the approval of the study protocol must also be provided in the manuscript.
