# Supplementary material for: Early contact between late farming and pastoralist societies in southeastern Europe
Source: Nature. 2023 Jul 19;620(7973):358–65. doi: 10.1038/s41586-023-06334-8 (PMC10412445; doi:10.1038/s41586-023-06334-8)
Supplement: Supplementary file 4 — This file contains legends for Supplementary Tables A–Y. [file 41586_2023_6334_MOESM4_ESM.docx]

**Supplementary Table A: General Information**

Excel file containing all general and meta information about the individuals included in this study, including amount of endogenous DNA after 1240k capture, X-contamination, mitochondrial data and Y-chromosome data.

**Supplementary Table B: Group labels**

Excel file containing information about published ancient and modern genomes used in the study. Additionally, it contains new group labels for published ancient groups and individuals that have been used in this study.

**Supplementary Table C: Map *f*_3_-statistics**

Excel file providing results of *f*_3_-statistics of the form *f*_3_(*test1, test2*; Mbuti) for all pairs of individuals for each time period.

**Supplementary Table D: Shared drift N CA and SEE N**

Excel file providing results for *f*_4_-statistics of the form *f*_4_(*test*, PIE039/CA; HGs, Mbuti) which tests for shared between PIE039/CA and different HG groups conditioned on Neolithic test populations.

**Supplementary Table E: Merged source populations**

Excel file showing the single groups that contribute to a merged population based on further *f*-statistics or archaeological information.

**Supplementary Table F: Shared drift PIE060 HGs**

Excel file containing results of *f*_4_-statistics of the form *f*_4_(SEE 3/SEE N, PIE060, HGs, Mbuti) which tests for shared drift between PIE060 and different HG groups conditioned on SEE 3 and SEE N.

**Supplementary Table G: distal qpAdm CA**

Excel file providing results for distal qpAdm modelling for PIE039 and CA groups.

**Supplementary Table H: proximal qpAdm CA**

Excel file providing results for proximal qpAdm modelling for PIE039 and CA groups.

**Supplementary Table I: READ**

Excel file providing READ results for all new individuals for each time period.

**Supplementary Table J: IBD sharing**

Excel file providing summary statistics of the IBD sharing, reporting both number and total length of IBD >8, 12, 16 and 20 cM, as well as longest IBD block, by the software ancIBD.

**Supplementary Table K: hapROH analysis**

Excel file providing inferred runs of homozygosity (ROH) using the software hapROH for all individuals with more than 400,000 SNPs on the 1240k SNP panel (n=73). For each individual, we report summary statistics of ROH, number and total sum of ROH longer than 4, 8, 12, and 20 cM, and the maximum ROH length.

**Supplementary Table L: ind attraction HGs, Turkey N**

Excel file providing results of *f*_4_-statistics of the form *f*_4_(*test*, Ukraine Eneolithic; *cornerstones*, Mbuti) as a test for shared drift between Ukraine Eneolithic individuals and *cornerstone* groups conditioned on Turkey_N, Steppe Eneolithic and Caucasus Eneolithic/Maykop

**Supplementary Table M: Steppe comparison**

Excel file providing results of *f*_4_-statistics of the form *f*_4_(*steppe1*, *steppe2*; Ukraine Eneolithic, Mbuti) as a test for shared drift between *steppe2* and Ukraine Eneolithic groups conditioned on *steppe1*.

**Supplementary Table N: group attr HGs, Turkey_N**

Excel file providing results of of *f*_4_-statistics of the form *f*_4_(*test*, Ukraine Eneolithic; *cornerstone*, Mbuti) as a test for shared drift between Ukraine Eneolithic groups and individuals and HG groups/Turkey_N conditioned on Turkey_N, Steppe Eneolithic and Caucasus Eneolithic/Maykop.

**Supplementary Table O: Immel et al. 2020**

Excel file providing results from the reanalysing of the published Cucuteni-Trypillia samples from Immel *et al.* 2020.

**Supplementary Table P: farmer attraction**

Excel file provoding results of *f*_4_-statistics of the form *f*_4_(Steppe Eneolithic/ Caucasus Eneolithic/Maykop, Ukraine Eneolithic; *test,* Mbuti) as a test for shared drift between Ukraine Eneolithic and farmer-related CA groups from SEE and Anatolia conditioned on Steppe Eneolithic and Caucasus Eneolithic/Maykop.

**Supplementary Table Q: HG attraction**

Excel file provoding results of *f*_4_-statistics of the form *f*_4_(Caucasus Eneolithic/Maykop, Ukraine Eneolithic; HGs, Mbuti) as a test for shared drift between Ukraine Eneolithic groups and individuals and HG groups conditioned on Caucasus Eneolithic/Maykop.

**Supplementary Table R: distal qpAdm Eneolithic**

Excel file providing results for distal qpAdm modelling for Ukraine Eneolithic groups and individuals.

**Supplementary Table S: proximal qpAdm Eneolithic**

Excel file providing results for proximal qpAdm modelling for Ukraine Eneolithic groups and individuals.

**Supplementary Table T: HG attraction EBA**

Excel file providing results of *f*_4_-statistics of the form *f*_4_(CA, EBA; HGs, Mbuti) as a test fo shared drift between EBA groups and HGs conditioned on their predecessor or contemporaneous group.

**Supplementary Table U: farmer attraction EBA**

Excel file providing results of *f*_4_-statistics of the form *f*_4_(*test*, EBA, VAR_CA, Mbuti) as a test for shared drift between EBA groups and VAR_CA as a farmer-related group conditioned on Yamnaya-associated groups.

**Supplementary Table V: Steppe Caucasus attraction**

Excel file providing results of *f*_4_-statistics of the form *f*_4_(Steppe Eneolithic/ Caucasus Eneolithic/Maykop, EBA; *cornerstones*, Mbuti) as a test for shared drift between EBA and Yamnaya-associated groups and *cornerstone* populations conditioned on Steppe Eneolithic and Caucasus Eneolithic/Maykop.

**Supplementary Table W: pairwise f4 Yamnaya**

Excel file providing results of *f*_4_-statistics of the form *f*_4_(*steppe1*, *steppe2*; *test*, Mbuti) as a test for shared drift between different Yamnaya-associated groups and various *test* groups.

**Supplementary Table X: distal qpAdm EBA**

Excel file providing results for distal qpAdm modelling for EBA groups.

**Supplementary Table Y: proximal qpAdm EBA**

Excel file providing results for proximal qpAdm modelling for EBA groups.
